# Sticking our nose into the Sonorini tribe: A new genus and species of snake (Squamata: Colubridae: Sonorini) from the Balsas Basin of Mexico

**DOI:** 10.1371/journal.pone.0337187

**Published:** 2025-12-10

**Authors:** Antonio Y. Cisneros-Bernal, Ricardo Palacios-Aguilar, Carlos Hernández-Jiménez, Eric N. Smith, Oscar Flores-Villela, Cristian Hernández-Morales, Oscar Olivares Loyola, Gustavo Campillo-García

**Affiliations:** 1 Posgrado en Ciencias Biológicas, Unidad de Posgrado, Circuito de Posgrados, Ciudad Universitaria, Coyoacán, Mexico; 2 Departamento de Biología Evolutiva, Facultad de Ciencias, Universidad Nacional Autónoma de México, Circuito exterior s/n, Ciudad Universitaria, Coyoacán, México; 3 Totlok A. C., Tlalpan, Mexico; 4 Facultad de Ciencias Biológicas, Benemérita Universidad Autónoma de Puebla, Puebla, Mexico; 5 Amphibian and Reptile Diversity Research Center, Department of Biology, The University of Texas at Arlington, Arlington, Texas, United States of America; 6 Argentine Dryland Research Institute of the National Scientific and Technical Research Council, Mendoza, Argentina; CONICET: Consejo Nacional de Investigaciones Cientificas y Tecnicas, ARGENTINA

## Abstract

The colubrid snake tribe Sonorini, which is largely composed of semifossorial and fossorial species, has undergone a series of taxonomic changes in the last few decades. New species have been added, multiple genera have been synonymized, and phylogenetic relationships have been tested using molecular systematics. Our field explorations of the dry Balsas Basin within the Mexican state of Puebla recently resulted in the procurement of two specimens of an unknown Sonorini species. Based on an integrative methodology of genetic and morphological data, we conclude that these specimens represent a hitherto unnamed genus and species, which we describe herein. This new genus is closely related to the other monotypic, Mexican endemic genera *Pseudoficimia* and *Sympholis*, but is easily diagnosable from them.

## Introduction

Mexico has the second-richest reptile biodiversity of any country in the world, surpassed only by Australia [1–3]. There are over 950 recognized Mexican reptile species, of which over 50% are considered endemic to the country [2,4]. Each year this number increases due to novel discoveries. From 2022 to 2024, ten new reptile species were described from Mexico [*e.g*., 5–13].

The remarkable diversity of reptiles in Mexico is attributable to multiple factors, but the main drivers of this diversification are generally considered to be the country’s climatic heterogeneity and complex geological history [14–17]. The combination of these abiotic factors has promoted extraordinary radiations in many animal groups, one of which being the snake family Colubridae. The species of this family occupy almost all available habitats in Mexico and display substantial morphological variability.

Small, elusive snakes in tropical regions present special challenges for researchers due to a combination of factors that include enigmatic behavior, small geographic ranges, and low population sizes [18]. Fossorial snakes spend most of their lives underground and hence are especially difficult to observe [19,20]. Within the Colubridae, 34 genera have been reported from Mexico [3], of which four (12%) are country endemics and have some degree of fossoriality or semi-fossoriality. These four endemic genera — *Conopsis*, *Geagras*, *Pseudoficimia*, and *Sympholis* — are considered part of an assemblage of burrowing snakes termed the “tribe Sonorini” [19,21–31].

As defined by Dowling [19], the snake tribe Sonorini includes the genera *Chilomeniscus*, *Chionactis*, *Conopsis*, *Ficimia*, *Gyalopion*, *Sonora*, and *Stenorrhina*. Some authors have proposed the inclusion of other genera such as *Tantilla*, *Tantillita*, *Scolecophis,* and *Geagras* based on osteological data [23,24]. Furthermore, Hardy [32] considered *Pseudoficimia* to be related to *Conopsis* and *Ficimia* due to shared morphological characters. Recent large-scale molecular phylogenies have recovered the tribe Sonorini as a monophyletic group and as one of the few resolved clades of Neotropical colubrids [26,29,31,33–35]. However, higher-level relationships of the tribe remain unclear. The tribe Sonorini is sometimes considered related to “racer snakes” like *Mastigodryas* and *Drymoluber* [26], or to a plethora of other genera such as *Coluber*, *Drymobius*, *Drymarchon*, *Masticophis*, and *Salvadora* [31]. Additionally, the composition of the tribe remains controversial since some formerly recognized genera have been synonymized, and few works have tried to elucidate the phylogenetic relationships among these taxa [25,30].

The first comprehensive phylogenetic hypotheses for the tribe Sonorini was presented by Holm’s dissertation [25], based on external morphology, molecular data, and osteology. In this work, he established that the tribe is composed of a “*Ficimia* clade” that includes *Conopsis*, *Ficimia*, *Gyalopion*, *Pseudoficimia*, *Stenorrhina*, and *Sympholis*, and which is sister to a “*Sonora* clade” that includes *Sonora*, *Chilomeniscus,* and *Chionactis*; in turn, he resolved these two clades as sister to a third clade composed of *Scolecophis* and *Tantilla.* Notably, Holm [25] considered *Geagras* and *Tantillita* to be synonyms of *Tantilla*. Most of the information available on the tribe focuses on works that deal with only one genus, such as *Conopsis* [28,36], *Ficimia* [20,32,37], *Pseudoficimia* [38], *Tantilla* [39–42], and the “*Tantilla* clade” including *Geagras*, *Scolecophis*, *Tantilla*, and *Tantillita* [43]. Recent phylogenetic revisions of *Sonora, Chionactis*, *Chilomeniscus*, and *Procinura* also resulted in the synonymization of the latter three genera within *Sonora* [30,44]. We do not accept Holm’s (2008) hypothesis that synonymizes *Geagras* and *Tantillita* with *Tantilla,* because the proposal was based on limited morphological characters (absence of loreal and apical pits on dorsal scales), reduced sampling per genus (*Geagras* N = 13 and *Tantillita* N = 8), and no genetic information. We consider both genera as independent until new evidence confirms or detracts their distinctiveness; additionally, we follow Cox et al. [30] in considering *Chilomeniscus*, *Chionactis*, and *Procinura* as synonyms of *Sonora*. Hence, we consider the tribe Sonorini to be composed of the genera *Conopsis*, *Ficimia*, *Geagras*, *Gyalopion*, *Pseudoficimia*, *Scolecophis*, *Sonora*, *Stenorrhina*, *Sympholis, Tantilla*, and *Tantillita*.

While exploring the dry forests of the Balsas Basin in the southwestern part of the Mexican state of Puebla, we encountered two specimens of Sonorini snakes that were unassignable to any known genus of the tribe or any other snake taxon based on their distinctive morphological characters. This pair of puzzling specimens and their phylogenetic placement are the focus of this contribution.

## Materials and methods

### External morphology

The two novel specimens of Sonorini were found dead, collected and tissue was taken under the permission of the law that regulates the scientific collection issued by SEMARNAT/DGVS with number FAUT015, given to O.F.V. For comparisons we adhered to standard scale count protocols primarily following Myers [45], with ventral scales counted according to Dowling [46], and scale terminology in the loreal region following Savage [47]. Six qualitative and six meristic morphological characters were examined (Table 1). Snout–vent length (SVL) and tail length were measured with a metal ruler and rounded to the nearest millimeter (mm) for comparisons (see Description section). Catalog numbers and the number of individuals per genus examined for external morphology are provided in S1 Appendix. All measurements were taken using digital calipers (Mitutoyo 500-196-30 AOS) under a stereomicroscope (Zeiss 475002) and rounded to the nearest 0.1 mm.

**Table 1:** Selected morphological characters in the genera composing the tribe Sonorini. Diagnostic differences with *Yakacoatl* are in grey. ^*^Denotes qualitative characters, while ^º^meristic characters.

Genus	^*^ Rostral (contact)	^*^ Loreal	^º^ Supralabials	^º^ Infralabials	^º^ Dorsal scale rows at midbody	^º^ Ventrals	^*^ Anal scale	^º^ Subcaudals	^º^ Number of maxillary teeth	^*^ Hemipenis (form)	^*^ Calyces	^*^ Apical ornamentation
*Yakacoatl*	Internasals	Absent	7	6	17	154–156	Divided	35–36	12	Subcylindrical, slightly bilobed	Absent	
												Spinules
*Conopsis*	Internasals	Variable	7 (5)	6 (4–7)	17	120–145	Divided	29–39	12–14	Subcylindrical, slightly bilobed	Absent	Spinules
*Ficimia*	Frontal	Absent	5–8	5–8	17	126–157	Divided	26–44	13–17	Simple, papillated	Present	Calyces
*Gyalopion*	Internasals	Absent	6–7	6–7	17	122–140	Variable	20–37	10–15	Simple, slighlty bulbous	Present	Spinulated calyces
*Geagras*	Internasals	Absent	5	6	15	113–124	Divided	26–33	10–13	Simple, capitate	Present	Calyces
*Pseudoficimia*	Internasals	Absent	7	7	17	139–163	Divided	34–52	13–17	Simple, asymetrical	Present	Papilated calyces
*Sonora*	Internasals		6–7	6–8	13–15	152–189	Divided	32–48	11–14	Simple	Absent	Spinules
*Stenorrhina*	Internasals	Absent	7	7–8	17	136–183	Divided	24–50	13–14	Simple, bulbous	Absent	Flounces
*Sympholis*	Internasals	Variable	5	6–7	19	207–232	Single	14–24	11–13	Simple	Present	Enlarged calyces
*Scolecophis*	Internasals	Present	7	6–7	15	181–198	Divided	45–54	13–14	Simple	Absent	Spinules
*Tantilla*	Internasals	Absent	6–7	6	15	106–197	Divided	19–85	12–20	Simple	Present	Calyces
*Tantillita*	Internasals	Absent	7	6	15	103-125	Divided	28-56	22–25	Simple	Present	Calyces

### Internal morphology

We identified sex through a small incision at the base of the tail. The retracted hemipenis of the holotype was prepared following the procedures of Myers and Cadle [18], and we cut the retractor muscle close to the attachment with the hemipenial lobes as suggested by Smith and Ferrari-Castro [48]. Terminology used in the hemipenial description and the diagnosis follows Dowling and Savage [49], and Myers and Campbell [50]. We applied the same methods for other comparative material revised. We prepared and/or revised 17 hemipenes representing 13 Sonorini taxa, including our novel specimens. We removed the left maxilla of the holotype to describe the maxillary arch and count teeth.

Additionally, we CT-scanned the head of the holotype and paratype specimens at the UTA Shimadzu Center for Environmental Forensics and Material using the Shimadzu inspeXio SMX-100CT scanner at a resolution of 12 μm with x-ray operating at 65 kV and 40 mA. The segmentation and the 3D models of the CT-scan were made in the software 3DSlicer 5.0.2 [51]. We revised 35 CT-scans available from Morphosource and other published sources and scanned six additional specimens, including our Sonorini specimens, for this work (S3 Table S1). The image stack of this CT-scan is available at www.morphosource.org The osteological terminology follows Cundall & Irish [52].

Examined comparative material can be consulted at Appendix 1. All museum acronyms follow Sabaj [53,54] with the addition of UAGRO for Laboratorio Integral de Fauna Silvestre, Universidad Autónoma de Guerrero.

Additional data on external morphology, hemipenial characters, and osteology were obtained from the following sources: Cox et al. [30,44], Goyenechea [28], Goyenechea and Flores-Villela [27,36], Hardy [20,32,38,55,56], Humphrey and Shannon [57], Mahrdt et al. [58], McCranie [59], Wilson [60,61], and Wilson and Mata-Silva [43] (Table 1).

### Molecular methods

*DNA extraction and PCR protocol*. We extracted genomic DNA of the two novel specimens of Sonorini using the DNeasy Blood & Tissue Kit protocol from Qiagen (Qiagen Valencia, CA) following the manufacturer protocol. We sequenced three mitochondrial loci including the small (*12S*) and large (*16S*) ribosomal units, and cytochrome b (*cytb*), together with the nuclear locus oocyte maturation factor MOS (*c-mos*), using the primers and PCR protocols described in Jadin et al. [62]. The sanger sequencing process was conducted in the Laboratorio de Secuenciación Genómica de la Biodiversidad (IB-UNAM).

*Phylogenetics methods.* We assembled forward and reverse reads of the resulting electropherograms in Geneious Prime 2021.0.3 (https://www.geneious.com). To assess the phylogenetic position of the two new Sonorini specimens, we downloaded available NCBI GenBank sequences from all the genera that are currently recognized within the tribe Sonorini including (N = number of taxa included): *Conopsis* (N = 3), *Ficimia* (N = 2), *Gyalopion* (N = 2), *Pseudoficimia* (N = 1), *Scolecophis* (N = 1), *Sonora* (N = 6), *Stenorrhina* (N = 2), *Sympholis* (N = 1), and *Tantilla* (N = 17), as well as representatives of 26 other Neotropical Colubrid genera to explore the phylogenetic affinities of the tribe Sonorini (S2 Table). We included as outgroup sequences the following snake families (number of terminals in parentheses): Acrochordidae (1), Aniliidae (1), Atractaspidae (1), Boidae (2), Calamariidae (1), Dipsadidae (4), Elapidae (1), Homalopsidae (1), Natricidae (1), Pareatidae (1), Psammophiidae (1), Pseudoxenodontidae (1), Tropidophiidae (1), Viperidae (2), and Xenodermidae (2). Whenever possible, we avoided mixing sequences of different individuals in our concatenated multi-locus dataset and abstained from using this kind of out available in repositories (S2 Table). The final dataset consisted of 94 terminals from 93 species, of which 37 are sonorines. We aligned most sequences using Muscle [63] and manually edited them by eye using PhyDE v.0.9971 [64], searching for conserved sites and adding gaps across the sequences when the alignment algorithm failed. The *12S* and *16S* sequences were aligned using MAFFT v.7 under the E-INS-i strategy [65].

We created a partition file specifying the partitions by codon and gene, as well as the best substitution model selected using Partition Finder v.2.1.11 [66] under the “greedy” algorithm and the Bayesian Information Criterion [67]. A Maximum Likelihood (ML) analysis was conducted with IQTree software [68], and node support was assessed using 5,000 UltraFast Bootstrap (UFB) replicates [69]. We visualized and edited the resulting trees in FIGTREE v.1.4.4 [70] and calculated uncorrected pairwise genetic distances (*p*-distances) for the aligned mitochondrial gene fragments of the sampled sonorines with MEGA X software [71].

***Nomenclatural Acts*.** —The electronic edition of this article conforms to the requirements of the amended International Code of Zoological Nomenclature, and hence the new names contained herein are available under that Code from the electronic edition of this article. This published work and the nomenclatural acts it contains have been registered in ZooBank, the online registration system for the ICZN. The ZooBank LSIDs (Life Science Identifiers) can be resolved and the associated information viewed through any standard web browser by appending the LSID to the prefix ““http://zoobank.org/”“. The LSID for this publication is: urn:lsid:zoobank.org:pub:C639D697-067A-4007-B891-BFC8F402FBBD. The electronic edition of this work was published in a journal with an ISSN, and has been archived and is available from the following digital repositories: PubMed Central, LOCKSS, Totlok.mx and bidi.unam.mx.

## Results

### Phylogenetic relationships

**Fig 1:**
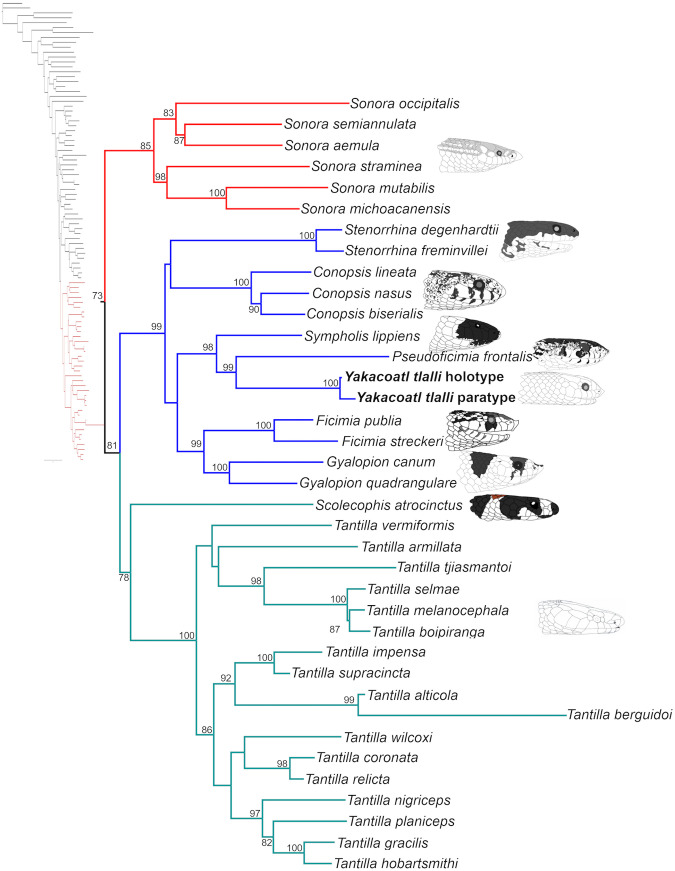
Phylogenetic relationships of the tribe Sonorini. Numbers at the nodes represent ultrafast bootstrap values. Heads at the tips are intended to be illustrative and are not at the same scale.

Our final genetic matrix consisted of 2794 base pairs (bp) and 282 sequences, including 530 bp for *12S* (57 sequences), 564 bp for *16S* (64), 1117 bp for *cytb* (82), and 583 bp for *c-mos* (79). Seven new sequences were generated for our new Sonorini specimens (S2 Table). We obtained and implemented the following partition scheme and substitution models for the ML analysis: *i*) *12S*, *16S*, *cytb* 1^st^ and 2^nd^ positions, and the three positions of *cmos* (GTR + I + G), and *ii*) *cytb* 3^rd^ position (K81UF+I + G). The resulting ML tree had a value of -lnL = –44862.998 (Fig 1; S1 Fig). The tribe Sonorini was recovered as monophyletic, though with low support (UFB 73%). No unambiguous sister clade to the tribe Sonorini was identified, although a weak relation (UFB 52%) was recovered to a poorly supported clade (UFB 52%) that includes *Chironius*, *Ptyas*, *Dendrophidion*, *Drymobius*, *Rhinobotrium*, *Leptodrymus*, *Palusophis*, *Drymoluber*, *Simophis*, and *Mastogodryas*. Within the tribe Sonorini, three main monophyletic clades were recovered that match the *Ficimia*, *Tantilla*, and *Sonora* clades proposed by Holm [25], with UFB support values of 99%, 78%, and 85%, respectively. The *Sonora* clade consisted solely of the genus *Sonora* and was divergent from a moderately supported clade (UFB 81%) that included the *Ficimia* and *Tantilla* clades. The *Ficimia* clade included *Conopsis*, *Ficimia*, *Gyalopion*, *Pseudoficimia*, *Stenorrhina*, *Sympholis*, and our two unknown Sonorini samples. All genera were retrieved as monophyletic and strongly supported (UFB 99–100%), but the relationships between them were poorly supported (>60%), except for two subclades: *i) Ficimia *+ *Gyalopion* (99%), and *ii*) a clade including *Sympholis*, *Pseudoficimia*, and the unknown Sonorini samples (98%). Our new specimens formed a monophyletic clade (UFB 100%) sister to *Pseudoficimia frontalis* (UFB 99%), which together are sister to the monotypic *Sympholis lippiens* with strong support (UFB 98%). This clade, in turn, was related to *Gyalopion* and *Ficimia*, albeit with low support (UFB 60%). The *Tantilla* clade groups *Scolecophis* and *Tantilla* with low support (UFB 78%), and recovers the genus *Tantilla* as monophyletic (UFB 100%).

Uncorrected pairwise distances of the three sequenced mitochondrial gene fragments were calculated (S4–S6 Table), although not all terminals were included for all loci due to incomplete sampling. For the *12S* gene (S4 Table), the genetic distances of the Sonorini *species inquirenda* holotype (MZFC 37100) ranged from 10.61% against its sister taxon *Pseudoficimia frontalis* to 17.42% against *Tantilla vermiformis*. For the *16S* gene (S5 Table), genetic distances of our Sonorini *species inquirenda* samples ranged from 5.53% against *Gyalopion quadrangulare* to 11.06% against *Sonora occipitalis*, and a distance of 6.28% from their sister group, *Pseudoficimia frontalis*. For the *cytb* gene (S6 Table), genetic distances of our Sonorini *species inquirenda* samples ranged from 12.04–12.25% against *Gyalopion canum* to 18.27–18.43% against *Tantilla wilcoxi*; genetic distances with their sister groups ranged from 13.5–13.85% against *Sympholis lippiens* to 15.46–16.06% against *Pseudoficimia frontalis*.

### Systematic account

Our morphological comparisons (see below, Table 1) support the phylogenetic analyses, and we consider that the snake specimens from the Balsas Basin of Puebla represent a new taxon member of the tribe Sonorini. Hence, a new genus and species are named herein:

***Yakacoatl* gen. nov.**

urn:lsid:zoobank.org:act:A1969E43-DBA2–4398-93B1-8416BBC0A505

Type species. ***Yakacoatl tlalli***, new species (described below).

***Diagnosis***.—This genus differs from all the other Western Hemisphere colubrids by the following set of characters: 1) smooth dorsal scales arranged in 17-17-17 rows, 2) an upturned rostral scale in contact with the internasals, 3) 154–156 ventral scales in known males; 4) 35–36 paired subcaudals scales; 5) short, thick supranasal bones; 6) absence of a supraoccipital keel; 7) 12 maxillary teeth, with rear teeth enlarged and faintly grooved; 8) hemipenis subcylindrical with slightly protruding lobes, a single *sulcus spermaticus*, body covered with spines and spinules, and lacking calyces.

This is considered a polythetic group and not a single character is identified as an autapomorphy. However, the combination of short, thick supranasal bones, absence of a supraoccipital keel, and lack of calyces on the hemipenis represent a unique condition within the Sonorini tribe.

*Comparison with known genera of Sonorini.*—*Yakacoatl* gen. nov. differs from *Geagras, Scolecophis, Sonora, Tantilla,* and *Tantillita* by having more dorsal scale rows, 17 (in comparison with 13–15), and from *Sympholis* by having fewer dorsal scales, 17 (19). It has more ventral scales than *Conopsis* and *Gyalopion*, 154–156 (in comparison with 120–145) but fewer than *Sympholis* and *Scolecophis* (181–232). It differs from *Ficimia, Gyalopion, Pseudoficimia, Stenorrhina* and *Tantilla* by the absence of calyces in the hemipenis (presence) (Table 1). Additional differences between *Yakacoatl* and the remaining Sonorini genera follows, character states of the new genus are in parentheses.

The monotypic genus *Pseudoficimia* is distinguished by having an elongated premaxillae that enters well between the paired elongated nasals (premaxillae not inserting that far and short, thick), 13–17 maxillary teeth (12), a conspicuous parietal ridge (absent), and a moderate median keel and straight anterior border of the supraoccipital (median keel poor, concave border). Additionally, the dorsal pattern in *Pseudoficimia* consists of conspicuous blotches (patternless or nearly so), it presents 7 infralabials (6), and the hemipenis has apical calyces and an asymmetrical apex (calyces absent, apical region with paired symmetrical projections; Fig 4).

**Fig 2:**
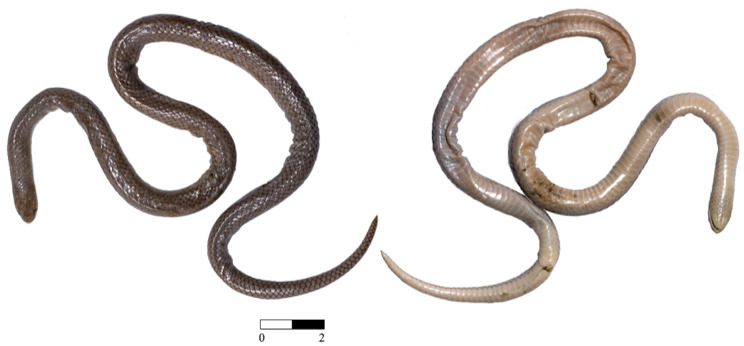
*Yakacoatl tlalli* (holotype, MZFC-HE 37100, 381 mm total length), dorsal (left) and ventral (right) aspects of the preserved specimen. Note the difference in size between the dorsal scales of the body and those from the tail. The scale bar represents centimeters.

**Fig 3:**
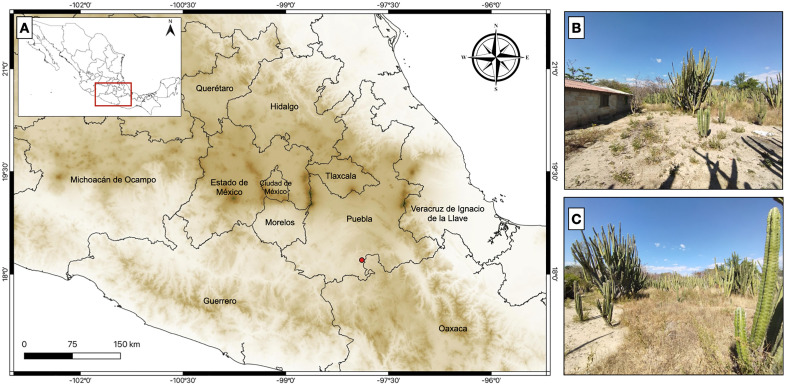
Location of the *Y. tlalli* type locality (red dot, A). Rural construction where the holotype (MZFC-HE 37100) was found (B); typical vegetation at the type locality (C). Photographs of the type locality were taken by OOL, shape files used to construct the map were obtained from INEGI (Instituto Nacional de Estadística y Geografía, Mexico).

*Sympholis* is a Mexican endemic and monotypic genus that represents one of the largest Sonorini, attaining an SVL of up to 540 mm (381 mm in only known adult male), and also has a banded dorsal pattern (patternless or nearly so) and a rounded snout (pointed snout). Furthermore, in *Sympholis* the premaxilla has a pointed rostrum (round) and two large lateral processes (small), the elongated nasals are curved downward at their lateral margin (short nasals with lateral margin directed laterally), there is an evident dorsolateral ridge on the parietal (absent), the postorbital is absent or small (present), there is a moderate median keel on the supraoccipital (poorly developed), the ectopterygoid is simple (bifurcated), it has a single anal plate (divided), 14–24 subcaudals (35–36), and the hemipenis is calyculated and simple (spinulate, slightly bilobed).

*Ficimia* species usually present a banded dorsal pattern, although *F. olivacea* has no dark markings on the dorsum (no conspicuous dorsal pattern). *Ficimia* is further differentiated from *Yakacoatl* by the contact of the rostral scale with the frontal (rostral contacting internasals). Additionally, the revision of available CT-scans of *F. publia*, *F. streckeri,* and *F. olivacea* (Supplementary material, S1 Table) reveals osteological differences such as articulation of the nasals with the frontals (separated), long and wide postorbital bones almost reaching the lower margin of the orbit (relatively short and thin postorbital bones), an ample supraoccipital bone (narrow), and a narrow anterior process of the cephalic condyle of the quadrate shaped like a narrow scythe (wide scythe or wide triangle). The hemipenial morphology of *F. publia*, *F. olivacea*, and *F. variegata* shows the presence of papillated calyces on a simple organ (calyces absent and slightly bilobed).

*Geagras redimitus* (monotypic) is a very distinctive snake that differs from *Yakacoatl* by having a very conical head (normal, anterior section of parietal relatively wide); premaxilla with a conical rostrum (short and wide) without lateral processes (with rounded lateral processes); a very ample articulation between nasals and frontals (not articulating); septomaxilla with long conchal processes (short) that are taller posteriorly (medially) and articulate with prefrontal and frontal; very short prefrontals (relatively high); ectopterygoid with very short anterior processes (moderately long) and a terminally truncated anterolateral process (slender and terminally pointed); supratemporal Y-shaped with one longer arm directed anterodorsally (bar-like); and quadrate short (tall). Additionally, *Geagras* can be differentiated from *Yakacoatl* by having 5 supralabials (7), 15 dorsal scale rows (17), 113–124 ventrals (154–156), 26–33 subcaudals (35–36), by lacking an upturned rostral scale (present), and by having hemipenes that are simple (slightly bilobed), capitated (uncapitated), and with calyces (absent).

*Gyalopion* is differentiated by its unique premaxilla, with a rostrum having an extensive flat anterior projection that is even laterally expanded, before the round lateral processes (premaxillary rostrum with only moderate projection and not laterally expanded). It also has a long and somewhat robust postorbital (relatively short and thin), a median keel on the supraoccipital (poorly developed), a very short and narrow scythe-like anterior process of the cephalic condyle of the quadrate (wide scythe or wide triangle of moderate length), 122–140 ventral scales (154–156), and a simple and slightly bulbous hemipenis with apical calyces (slightly bilobed organ without calyces).

Both species of *Stenorrhina* have the ectopterygoid with a short, expanded and terminally truncated anterolateral process (slender, long and terminally rounded), a very short triangular anterior process of the cephalic condyle of the quadrate (wide scythe or wide triangle of moderate length), two mental foramina on the dentary (one), and three foramina below prefrontal on the anterior portion of the maxillae (one anterior to the prefrontal). *Stenorrhina* further presents a characteristic fusion of the internasal scales with the nasal (separated), 7–8 infralabials (6), and the hemipenes being simple, bulbous, and covered with flounces at the apical region (simple, slightly bilobed, covered with spines and spinules).

Available CT-scans of *Conopsis* (*C. biserialis*, *C. nasus*, *C. sp.*; Supplementary material, S1 Table) show the premaxilla presenting a narrow rounded rostrum (wide), dorsally expanded postorbital bones (thin), and the presence of a well-developed median keel on the supraoccipital (poorly developed). Additionally, *Conopsis* spp. have 120–145 ventral scales (154–156), and lack an upturned rostral scale (present).

Representatives of *Sonora* (based on CT-scans of *S. cinctus*, *S. occipitalis*, *S. semiannulata*, and *S. straminea*; Supplementary material, S1 Table) present a well-developed premaxilla (not conspicuously enlarged), postorbital bone contacting frontal, and compound bone with prearticular crest noticeably higher than surangular crest (subequal). *Sonora* can also be distinguished from *Yakacoatl* by having 13–15 dorsal scale rows at midbody (17).

*Scolecophis* is a slender snake (stout), with an elongated head (short), frontal bones (rectangular), and braincase (short); premaxilla without anterior rostral projection (present); premaxillary ascending process thin throughout (wide at base), vertical (slanted backwards), and not separating nasals (separating); postorbital bone in broad contact with frontal (not in contact); and ectopterygoid with a short, expanded and terminally truncated anterolateral process (slender, long and terminally rounded). Additionally, *Scolecophis* has a tricolored dorsal body pattern resembling that of coral snakes (patternless or nearly so), 15 dorsal scale rows (17), 181–198 ventrals (154–156), and 45–54 subcaudals (35–36). *Tantilla* presents a vast amount of variation in many morphological characters, and to date, a comprehensive phylogenetic hypothesis of this genus is lacking as well as an exhaustive review of the characters exhibited by the 69 described species [3]. The premaxillary ascending process of *Tantilla* is variable, from thin to wide, from vertical to directed backwards and sometimes fitting between the nasals anteriorly but, even with this last condition, direct dorsal articulation between the ascending process and the nasals is not observed in our samples (in broad contact). Additionally, all *Tantilla* can be differentiated from *Yakacoatl* by having 15 dorsal scale rows (17), by lacking an upturned rostral scale (present), and having calyces present in the hemipenes (absent).

*Tantillita* (CT-scan of *Tantillita lintoni* available) differs from *Yakacoatl* by having the ascending process of the premaxilla not contacting or being situated between the nasals (in broad contact), postorbital bone in broad contact with frontal (not in contact), and ectopterygoid with an expanded and terminally truncated anterolateral process (slender, long and terminally rounded). Additionally, *Tantillita* can be differentiated from *Yakacoatl* by having 15 dorsal scale rows (17), by lacking an upturned rostral scale (present), and having calyces present in the hemipenes (absent).

***Etymology.***—From the Nahuatl words *yacatl* meaning nose, and *coatl* meaning snake, in reference to the pronounced rostral scale that resembles a pointed nose in the species. Of feminine gender.

***Yakacoatl tlalli* sp. nov.**

**Holotype.**—MZFC-HE 37100 (Fig 2), adult male from San Jerónimo Xayacatlán, Santo Domingo Tonahuixtla Municipality, Puebla, Mexico, 1335 m elevation (18.20517ºN, 97.89025ºW), collected 7 November 2014 by Oscar Olivares Loyola in a crop field (Fig 3B).

**Paratype.**—UTA R-66192, juvenile male from San Jerónimo Xayacatlán, Municipio Santo Domingo Tonahuixtla, Puebla, Mexico 1341 m elevation (18.20617ºN, 97.88856ºW), collected 19 May 2015 by Oscar Olivares Loyola in a crop field.

**Fig 4:**
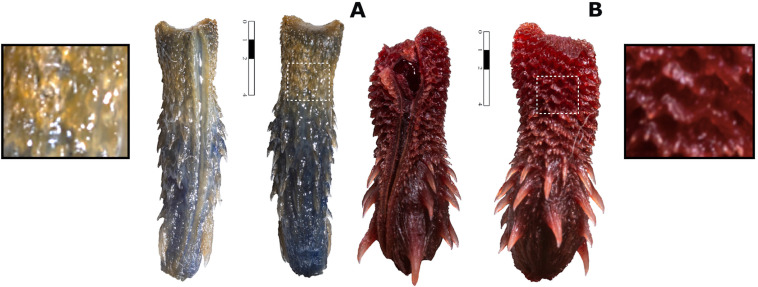
Hemipenis of *Yakacoatl tlalli* (A) and *Pseudoficimia frontalis* (B), a closely related Sonorini according to our phylogenetic tree. Sulcate and asulcate views are presented as left and right, respectively. Scale bar represents millimeters.

**Fig 5:**
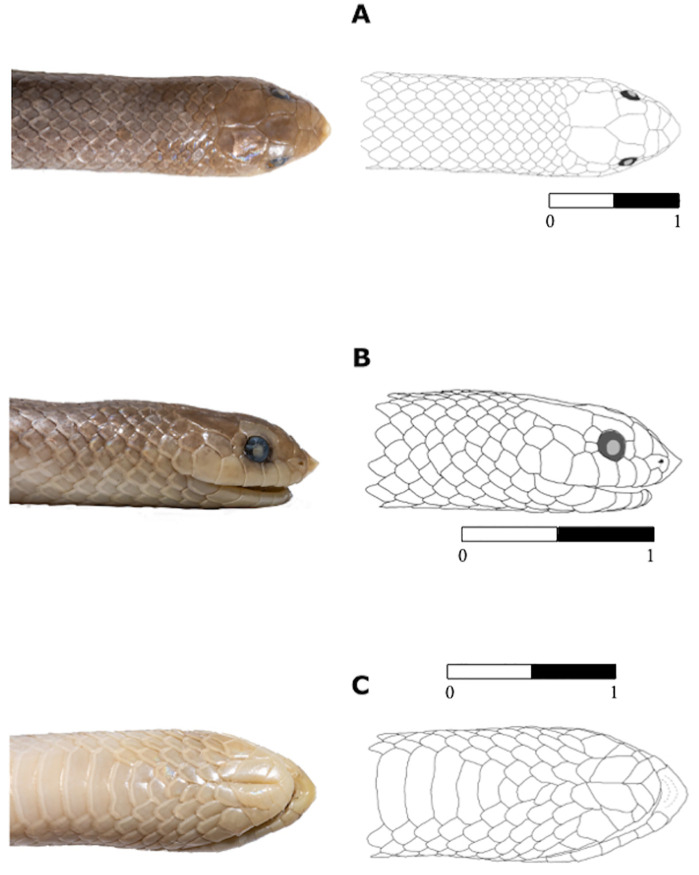
Dorsal (A), lateral (B), and ventral (C) views of the head of the holotype of *Yakacoatl tlalli* (MZFC-HE 37100). Scale bar represents millimeters.

***Description of the holotype***.—Adult male, judged by the presence of mineralized spines on hemipenis (Fig 4); specimen in fair condition, except for some small wounds on the ventral surface. SVL length 319 mm, tail length 62 mm; head not distinct from body, 10.1 mm long, 6.3 mm wide (Fig 2); eye diameter 1.4 mm; snout length 3.4 mm; dorsal arrangement of head plates in classic colubroid fashion (Fig 5); upturned rostral, 1.4 mm long, 2.6 mm wide; two paired internasals, 1.1 mm tall, 1.7 mm wide, 0.4 mm contact; two paired prefrontals, 1.3 mm tall, 2.2 mm wide; frontal longer than wide (3.6 x 2.7 mm), bordered by two supraoculars, and paired parietals, 4.4 mm long, 3.1 mm wide, 2.8 mm contact, longer and shorter than frontal; rostral contacting nasal, internasals, and first supralabials; nasal divided below, in contact with prefrontal and first and second supralabials; no loreal; one preocular; two postoculars; temporals 1 + 2 on right side, apparent malformation on “upper” secondary temporal, 1 + 1 on left side; seven supralabials, the first noticeably shorter than the others, 3–4 contacting orbit; six infralabials, first pair in ample contact, 1–3 contacting first pair of chinshields; two pairs of chinshields, first pair 2.9 mm tall and 1.6 mm wide, second pair 1.8 mm long and 1.4 mm wide; five rows of gulars; dorsals in 17-17-17 rows, smooth, lacking apical pits except for some fine marks in some scales; anal ridges absent; six scale rows at middle of tail; scales with uniform size throughout body, but becoming noticeably enlarged towards the rear and at level of cloaca; ventral scales 154; anal plate divided; subcaudal scales paired, 35 + cornified terminal spine; the dorsal coloration is smoke-grey, with enlarged scales at the level of the cloaca, surrounded by black outlines; the ventral part is creamy white.

***Hemipenes***— *In situ* right organ covered by transparent sheath with few melanized reticules; organ reaches end of subcaudal 10, bifurcates dextrally at middle of subcaudal 9 and medially at anterior of subcaudal 10; retractor muscle inserts at level of subcaudals 28 and 29. *Ex situ* organ (Fig 4A) is simple and subcylindrical in shape, except for two paired projections at apex; *sulcus spermaticus* undivided, bordered by definite lips, ending next to right projection of apex; sulcate ornamentation from base to apex: pedicel essentially naked, followed by two large basal spines decreasing in size towards apex, region between apical projections wide and naked; ornamentation on asulcate side as in sulcate.

**Fig 6:**
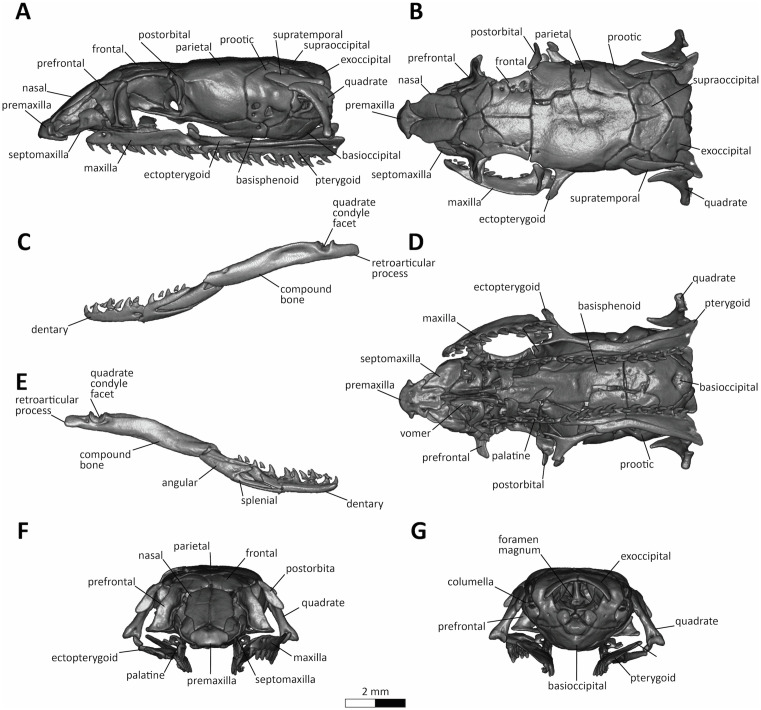
CT-Scanned skull of the holotype (MZFC-HE 37100). Lateral (A), dorsal (B), ventral (D), anterior (F), and posterior (G) view of the skull; lateral view of the left (C) and right (E) lower mandible. Scale bar represents millimeters.

***Osteology*** —Skull of holotype (Fig 6) presents some fractures on frontals, parietals, supraoccipitals, and compound bones, but overall condition allows detailed osteological observation; teeth counts given as left/right; ascending process of premaxilla with a broad base, projects posterodorsally separating nasals anteriorly; premaxilla presenting a short and wide rounded rostrum with rounded lateral processes, short transverse processes, a prominent vomerine process with parallel margins and bilobated end; nasals acute anteriorly and posteriorly, wider medially, separated from frontal; frontal paired, squarish, anterior border with soft slit, lateral border medially curved, interolfactory pillar present, three foramina laterally, supraorbital ridge present; parietal truncated anteriorly, contacting frontal, midpart globous, posterior border rounded, bending anteroventrally to basisphenoid; postorbital contacting parietal near frontoparietal suture, projecting ventrally to level of mid-orbit, compressed anteroposteriorly; supratemporal laminar, bar-like, contacting prootic anteriorly and quadrate posteriorly; septomaxilla narrow anteriorly, wider posteriorly, conchal process short and with broad rounded end directed dorsally at midlength and not articulating with prefrontal or frontal; vomer dorsally concave, posterior border with laterally compressed palatine process; prefrontal formed by facial sheet anteriorly, orbitonasal flange posteriorly, vertical prominent anteorbital ridge; orbitonasal flange pierced by a rounded and wide lacrimal foramen; quadrate compressed laterally, cephalic condyle expanded and shaped like a wide scythe directed anteriorly, mandibular condyle narrow, stylohyal process present as oval disc on medial surface of quadrate; supraocciptal roughly heart-shaped, lacking transverse crests and having a poorly developed median keel; prootic with three ventrolateral foramina, maxillary branch of trigeminal nerve with two anterior exits, dorsal bigger than ventral, mandibular branch of trigeminal opening posterolaterally; exoccipital divided, oval foramen opens posteriorly, recessus scalae tympani located ventrally to oval foramen; basisphenoid lacking parabasisphenoid keel; stapes with prominent footplate inside the inner ear cavity and not visible externally, shaft robust and projecting posteriorly to reach middle of cephalic condyle of quadrate.

Palatomaxillary arch with three dentigerous bones, maxilla, palatine, and pterygoid; maxilla starts at level of second supralabial, ends at level slightly behind eye, with soft dorsolateral curvature, one anterior facial foramen; maxillary teeth 12/12, heterodont, gradually enlarging towards rear, last three slightly grooved laterally; ectopterygoid Y-shaped, expanded anteriorly to embrace posterior end of maxilla, anteromedial process slender and pointed, anterolateral process slender and rounded at tip, contacting pterygoid posteriorly; palatine with anterior maxillary process expanded dorsolaterally, harboring large fenestra parallel to main axis, choanal process expanding dorsomedially, from mid-palatine and tip curving ventrally, teeth 7/8, nearly homodont; pterygoid partially sutured along length between dentary surface and lateral projection embracing ectopterygoid, expanded posteriorly, teeth 13/13, nearly homodont throughout; dentary bowed anteromedially, single mental foramen at middle of lateral face, teeth 13/13; splenial bar-like, pointed anteriorly, expanded posteriorly, fits into slit at medial surface of dentary; angular contacting splenial anteroventrally, coronoid process of dentary dorsally; compound bone overlapping and fitting into dentary anterodorsally, adductor fossa extending from midlength to articular facet for quadrate, prearticular crest subequal in height to surangular crest, concavity laterally below adductor fossa, retroarticular process laterally compressed and posteriorly projected.

***Variation in the juvenile male paratype (measurements in mm)***.—SVL 131, tail length 28, head length 6.35, head width 3.3, eye diameter 1.1, snout length 1.75; dorsal scale rows 17-17-17; ventrals 156; anal plate divided; subcaudals 36; supralabials 7/7; infralabials 7/7; preoculars 1/1; postoculars 2/2; scutellation characters as in adult holotype except for fourth infralabial on the right side barely reaching first pair of chinshields; coloration differs by presence of black mark below orbit, and faint striped pattern encompassing dorsal scale rows 3–4, 5–6, 11–12, 14–15.

**Fig 7:**
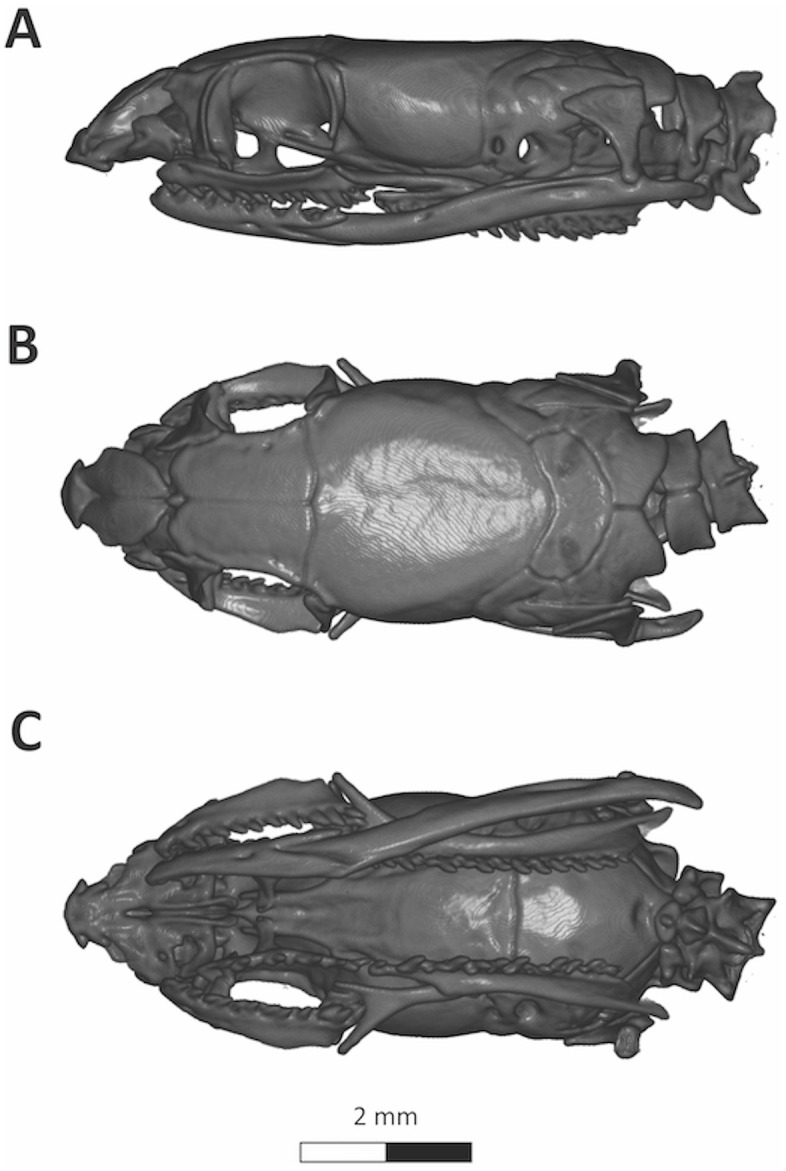
CT-Scanned skull of the paratype of *Yakacoatl tlalli* (UTA R-66192). Scale bar represents millimeters.

Skull of paratype (Fig 7) similar to that of the holotype, except for having slightly shorter nasals; a small azygous spherical bone between the anterior tip of the frontals; frontals with two foramina laterally; postorbital slightly longer and more slender, projecting ventrally to level of ¼ of orbit diameter below mid-orbit; supratemporal more acute anteriorly; quadrate slightly shorter and more robust, with anterior cephalic condyle triangular anteriorly; supraoccipital shorter, band-shaped; maxillary teeth 12/12, as holotype; palatine teeth 8/7, as in holotype; pterygoid teeth 15/14, slightly higher than holotype; dentary teeth 13/14, one count higher than in holotype.

**Fig 8:**
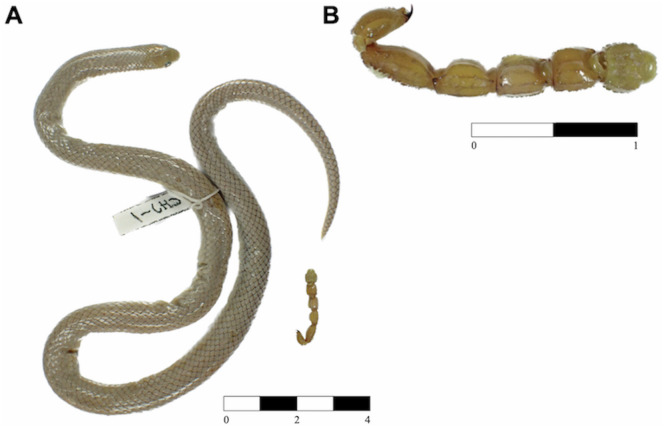
(A) Holotype of *Yakacoatl tlalli* (MZFC-HE 37100) with prey item. (B) Food item consisting of a scorpion tail of the genus *Diplocentrus* (Alacranidae) found in the stomach of the snake. Scale bars represent centimeters.

***Distribution and natural history***. — Both known specimens of *Yakacoatl tlalli* were obtained dead near the surroundings of San Jerónimo Xayacatlán, Puebla, Mexico, in the Balsas Basin. The snakes were obtained in a rural home, the holotype being preyed by a domestic chicken (*Gallus gallus*), and the paratype was found dehydrated at the same house (Fig 2B). Original vegetation at the type locality consisted of tropical deciduous forest but is currently heavily fragmented by agricultural activities. Upon dissection of the adult holotype, we obtained in its stomach the tail of a scorpion of the genus *Diplocentrus* (Alacranidae) (Fig 8).

***Etymology*.**—The specific epithet *tlalli*, comes from the Nahuatl word for land or earth. This naming choice alludes to the presumed fossorial habits of ***Yakacoatl tlalli***, inferred from its morphology and the known behavior of other Sonorini genera.

***Specimen identification using available keys*.**—Dichotomous keys for identifying the snake genera of Mexico and Central America are available in González-Hernández et al. [72], Heimes [73], Köhler [74], and Peters and Orejas-Miranda [75]. Running the known specimens of *Yakacoatl* through these keys would lead to the following couplets:

In Heimes [73], one would be led to couplet 58 with a choice between *Geagras*, *Tantilla*, and *Tantillita*, while in González-Hernández et al. [72], one would reach couplet 107 with a choice between *Tantilla* and *Tantillita*. See above for differences between known specimens of *Yakacoatl* and those genera. In Köhler [74], examination would lead to couplet 3, where one can choose between *Ficimia* and the dipsadid genus *Phimophis*; *Ficimia* is easily distinguishable by the contact of the rostral scale with the frontal (absent in *Yakacoatl*, see above for further comparisons), while *Phimophis* may be differentiated by the presence of a loreal scale (absent), 21-19-17 dorsal scale rows (17 throughout), and >190 ventral scales (154–156 [76–78]). In Peters and Orejas-Miranda [75], examination would lead to couplet 43, reaching the dipsadid genus *Enulius*; this genus and the related *Enuliophis* are differentiated by having extremely thin and long tails with >80 subcaudals (35–36), and an elongated loreal (absent) [59].

## Discussion

The semi-arid Balsas Basin is recognized as a region possessing high levels of regional and local endemism in a wide array of taxa [79–84], but also as a vulnerable ecoregion [79,81,82]. Due to the Basin’s biotic composition, two biogeographical units (termed *districts* by [85]) have been proposed, the Lower and the Upper Balsas Basin [85,86], with the latter being the origin of the type series of *Y. tlalli*. Previous authors have recognized the relevance of the Balsas Basin using herpetofauna as a model [14,87,88], and studies involving other taxonomic groups have highlighted its importance as a refuge during dramatic climatic events such as the Last Glacial Maximum (e.g., [83,85]). Although several endemic species have been reported from the Basin, it remains largely unexplored. According to recent checklists and our own data, there are at least 77 snake species distributed within the Balsas Basin in the states of Estado de México, Guerrero, Jalisco, Michoacán, Morelos, Oaxaca, and Puebla [89–93 and E. A. Reyes-Velázquez, unpublished]. However, the number of snakes reported from the Balsas Basin within each state ranges from 16 to 44, which is low considering that the number of reported snake species in these states can be as high as 166 in Oaxaca [91]. Additionally, there are very few species of snakes considered endemic to the Balsas Basin, namely *Coniophanes melanocephalus*, *Micrurus laticollaris*, *Thamnophis postremus*, and *Tropidodipsas zweifeli*. We now add *Yakacoatl tlalli* to the short list. Whether this low number of endemic snake species represents a sampling bias or the result of historical events remains an open question.

Given the morphology of *Yakacoatl*, we initially hypothesized a close relationship with *Conopsis* or *Pseudoficimia*. The grooved rear fangs and the lack of hemipenial calyces in *Yakacoatl* are similar to *Conopsis*, while its body size and high ventral scales count resemble *Pseudoficimia* (Table 1). However, our phylogenetic analysis suggests a close relationship between *Yakacoatl*, *Pseudoficimia,* and *Sympholis* (Fig 1). According to Holm [25], who used morphological characters and a fragment of the *cytb* gene, the sister taxa of *Sympholis* and *Pseudoficimia* were *Gyalopion* and *Stenorrhina*, respectively. These relationships were not supported in later molecular phylogenies, which instead suggested for the first time that *Sympholis* and *Pseudoficimia* were sister taxa [26,31]. Based on our work, *Yakacoatl* now joins *Pseudoficimia* and *Sympholis* as a third endemic, monotypic Sonorini genus distributed in seasonally dry forests along the Pacific drainages of Mexico.

We lack firsthand knowledge of the natural history of *Yakacoatl,* as both specimens were found dead. Nonetheless, characteristics of its cranium such as a modified premaxilla, short supratemporals, compact and short braincase, and a short occipital condyle suggest fossorial habits. The enlarged rear fangs and stomach content indicate a diet specialized in arthropods as in many Sonorini [23]. According to Jackson et al. [94], a diet including centipedes, spiders, and scorpions has been reported in *Sympholis*. Holm [25] has reported beetle larvae for this species and suggested a burrowing lifestyle and a probable interaction with *Atta mexicana* nests, including secretion of an oily substance that may protect the snake from bites. In the case of *Pseudoficimia*, minimal data on its natural history have been published. We have collected specimens from the Balsas Basin under vegetal debris, and a diet consisting of Lepidoptera larvae and tarantulas of the genus *Aphonopelma* have been reported for specimens collected in Sonora [95]. It is important to mention that we do not consider *Yakacoatl* to have any degree of anthropophilia. Despite having been found in areas of high disturbance, we believe that *Y. talli* individuals are burrowers and do not usually inhabit anthropogenic areas (as suggested by its only recent discovery). After the two specimens (holotype and paratype) were encountered, further field trips to the area in search of additional individuals were unsuccessful. Therefore, while there are genera that are highly tolerant to disturbances and have relatively high abundances (e.g., *Conopsis*), we believe that this is not the case for *Y. tlalli*. Based on the morphological evidence and the encounter locality, we believe that this species usually inhabits xerophilous zones of the area, buried under rocks or logs.

Although the tribe Sonorini has been repeatedly recovered as a monophyletic group, the distinctiveness of genera such as *Geagras* and *Tantillita* remains to be tested [25,43]. Although they have been proposed as invalid genera [25], we believe that there is insufficient evidence for that proposal, given the limited number of organisms from each genus analyzed and the plasticity of the characters that support synonymy. Their inclusion in phylogenetic analyses with genetic data should be a focus of future research. On the other hand, the validity of *Scolecophis* and *Tantilla* as another clade within the tribe, and the relationships of the former with other Colubridae, remain challenging (see [35] for a summary of these conflicting higher-level relationships). The taxonomy of the speciose genus *Tantilla* represents another challenge, and until very recently few efforts have been made to resolve the relationships within the genus [39–41]. Although this genus has received scant attention, it is considered one of the most diverse snake genera in the world [42,43,89], which counters the argument that the Neotropical fossorial snakes are an “evolutionary dead-end” [96].

To our knowledge, since the year 2000, at least 58 Mexican snake taxa have been resurrected, described, or elevated from subspecific to specific status (Supplementary material, S3 Table). Of these, 24 (~42%) represent fossorial/semifossorial species of the genera *Cenaspis, Chersodromus*, *Epictia*, *Geophis*, *Rena*, *Rhadinaea*, *Rhadinella*, *Tantilla,* and now *Yakacoatl*. Furthermore, most of these taxa were described based on the discovery of new populations previously unknown to science, which is a comparatively infrequent occurrence among Mexican squamate descriptions [97]. With the recognition of *Yakacoatl,* there are now 16 snake genera endemic to Mexico, and they represent 18% of the currently known snake diversity in the country.

## Supporting information

S1 Appendix Specimens examined, exclusive of CT-scanned material. See Materials and Methods for information on acronyms used. RPA refers to uncatalogued specimens to be deposited at the herpetological collection of the MZFC.
(DOCX)

S1 Fig Unedited tree resulting from the ML analysis of the Sonorini tribe. (PDF)

S1 Table Taxa and respective CT-scans examined for osteological comparison. Morphosource ID number provided. * Denotes specimens scanned for this study.
(DOCX)

S2 Table Species, specimen vouchers, and GenBank accession numbers used in our phylogenetic analysis. Taxa in bold represent members of the tribe Sonorini.
(DOCX)

S3 Table Mexican snake species listed with at least one taxonomic status modification since the year 2000 to date. (DOCX)

S4 Table Genetic uncorrected pairwise distances calculated from the 12S gene using MEGA X software. (DOCX)

S5 Table Genetic uncorrected pairwise distances calculated from the 16S gene using MEGA X software. (DOCX)

S6 Table Genetic uncorrected pairwise distances calculated from the cytb gene using MEGA X software. (DOCX)
