# Supplementary material for: Sticking our nose into the Sonorini tribe: A new genus and species of snake (Squamata: Colubridae: Sonorini) from the Balsas Basin of Mexico
Source: PLoS One. 2025 Dec 10;20(12):e0337187. doi: 10.1371/journal.pone.0337187 (PMC12694871; doi:10.1371/journal.pone.0337187)
Supplement: S1 Appendix — See Materials and Methods for information on acronyms used. RPA refers to uncatalogued specimens to be deposited at the herpetological collection of the MZFC. (DOCX) [file pone.0337187.s001.docx]

**S1 Appendix.** Specimens examined, exclusive of CT-scanned material. See Materials and Methods for information on acronyms used. RPA refers to uncatalogued specimens to be deposited at the herpetological collection of the MZFC.

*Conopsis acuta* (N= 1). MZFC 8323

*Conopsis biserialis* (N= 1). MZFC 3613.

*Conopsis megalodon* (N= 1). MZFC 24588.

*Conopsis nasus* (N= 1). MZFC 26331.

*Ficimia hardyi* (N= 6). MZFC 4876 (paratype), 4877 (holotype), 4878 (paratype), 5328 (paratype), 5348 (paratype), 20915.

*Ficimia olivacea* (N= 27). AMNH 19774, 19787–19788, 99140, 162104; USNM 6329, 30131, 224834–224837; TCWC 32909, 37717, 59955, 68154; UTA-R 1644–1651, 7915, 16128; MZFC 5059, 8525.

*Ficimia publia* (N= 51)*.* AMNH 65120, 65896, 66459, 67961, 88832; CAS 114049–114051, 154146; TCWC 21960; USNM 12688, 16427–16428, 55237–55238, 110295–110298, 121452, 123491–123492, 140124, 561034, 565807, 578325, 64986; UTA-R 3023, 3054–3056, 3726, 3735, 9998, 10327, 22164, 25376, 26576, 34997, 39213–39214, 41164; MZFC 379, 4221, 4665, 7083, 7768, 10875, 16509, 18428–18429.

*Ficimia ramirezi* (N= 1). UIMNH 3767, holotype.

*Ficimia ruspator* (N= 1). UIMNH 25064, holotype.

*Ficimia streckeri* (N= 95). AMNH 64026, 79093, 93416–93421, 99149, 110404–110406, 107282–107287, 126721–126722, 158658–158660; ANSP 14773, 20013; CAS 100075–100084; TCWC 10580, 27369, 31535, 33642–33644, 36368–36369, 38981, 49908–49911, 53050–53053, 56426, 56835, 58113, 58119, 58122, 62294, 81961, 84818, 85206; USNM 25201–25202, 101051, 125120, 238844, 244765, 299629, 307542, 321515; UTA-R 4677, 4857, 5705, 7023, 10325–10326, 15829–15830, 16606, 16698, 16776–16777, 16811–16812, 17182, 17791, 17955–17959, 37948; MZFC 9796, 13747.

*Ficimia variegata* (N= 2). CAS 140988, USNM 30126,

*Geagras redimitus* (N= 1). UTA R-26690.

*Gyalopion canum* (N= 1). MZFC 31841

*Gyalopion quadrangulare* (N= 4). MZFC 15243, 19506, 22388, 26843.

*Pseudoficimia frontalis* (N= 6). MZFC 21, 331, 12732, 12735, 26933–22934.

*Sonora michoacanensis* (N= 5). MZFC 17246, 23344, 23487, 26977; RPA 1310.

*Sonora straminea* (N= 1). MZFC 20314.

*Stenorrhina degenhardtii* (N= 1). MZFC 36519.

*Stenorrhina freminvillei* (N= 6) MZFC 34723; RPA 45, 1022, 1265-1266, 1364.

*Sympholis lippiens* (N= 1). MZFC 26859.

*Tantilla calamarina* (N= 38). CNAR 18729–735, 28923–28924, 29208-220; MZFC 2206, 13810–13815, 19793–19801, 35822, 35826.

*Tantilla carolina* (N= 1). BMNH 1906.6.1.241, holotype.

*Tantilla ceboruca* (N= 1). UTA-R 58516.

*Tantilla coronadoi* (N= 1). MZFC 25507.

*Tantilla deppii* (N= 3). MZFC 33747, 33820, 33822.

*Tantilla rubra* (N= 1). UTA R-12455.

*Tantilla shawi* (N= 1). UTA R-36810.
