# Supplementary figures and images for: Sticking our nose into the Sonorini tribe: A new genus and species of snake (Squamata: Colubridae: Sonorini) from the Balsas Basin of Mexico
Source: PLoS One. 2025 Dec 10;20(12):e0337187. doi: 10.1371/journal.pone.0337187 (PMC12694871; doi:10.1371/journal.pone.0337187)

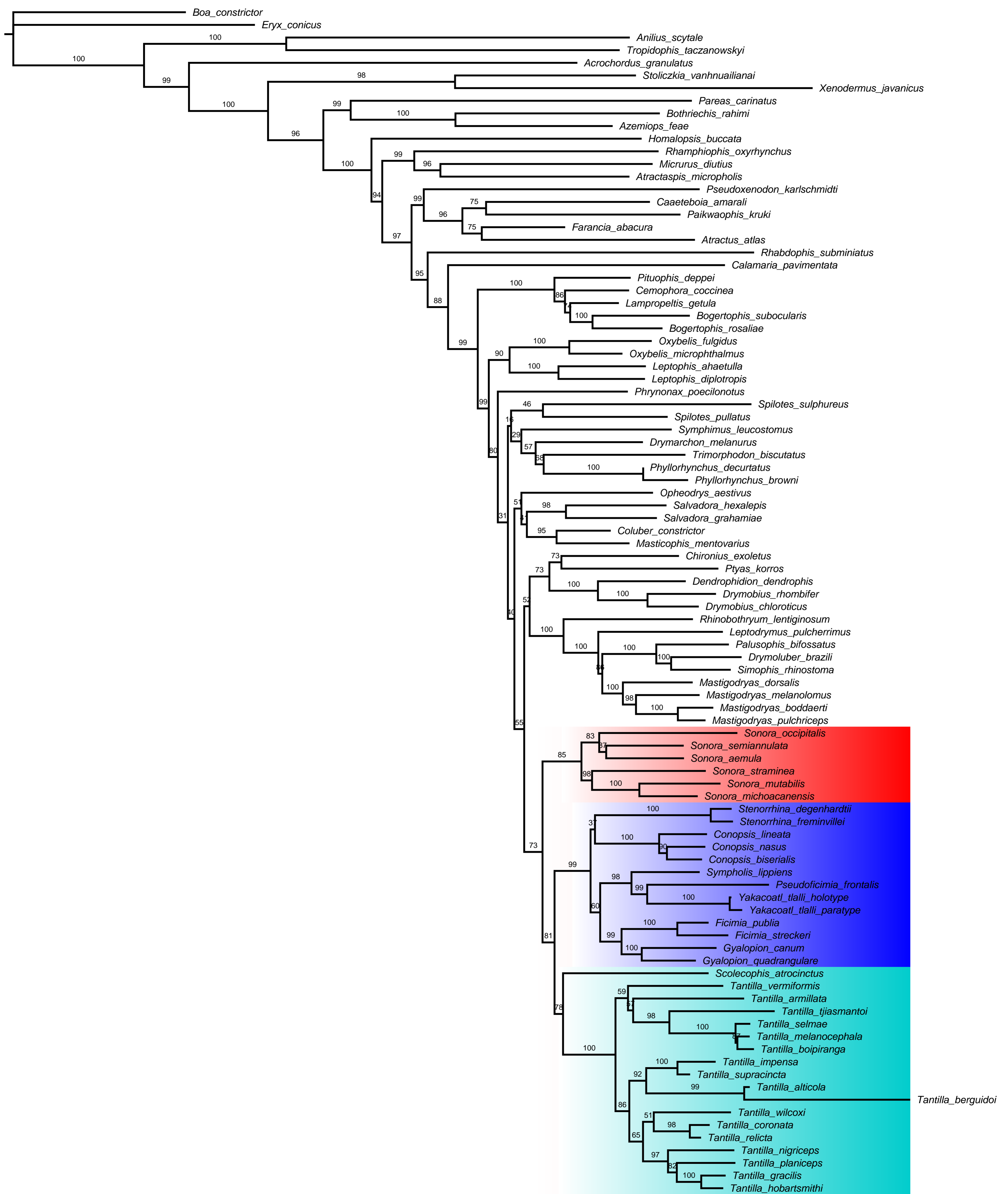

Supplement: S1 Fig — (PDF) [file pone.0337187.s002.pdf]
