# Supplementary material for: Sticking our nose into the Sonorini tribe: A new genus and species of snake (Squamata: Colubridae: Sonorini) from the Balsas Basin of Mexico
Source: PLoS One. 2025 Dec 10;20(12):e0337187. doi: 10.1371/journal.pone.0337187 (PMC12694871; doi:10.1371/journal.pone.0337187)
Supplement: S1 Table — Morphosource ID number provided. * Denotes specimens scanned for this study. (DOCX) [file pone.0337187.s003.docx]

**Table S1.**Taxa and respective CT-scans examined for osteological comparison. Morphosource ID number provided. * Denotes specimens scanned for this study. (DOCX).

| **Species** | **Catalog number** | **Morphosurce ID** |
| --- | --- | --- |
| *Arizona elegans* | UTA R-63119 | **XXXX*** |
| *Conopsis biserialis* | UMMZ 149711 | 0000S8178 |
| *Conopsis nasus* | MVZ 164712 | 000S22737 |
| *Conopsis sp* | UMMZ 128047 | 0000S9899 |
| *Geagras redimitus* | UTA R-26690 | **XXXX*** |
| *Gyalopion canum* | UMMZ 142551 | 000S11110 |
| *Gyalopion quadrangulare* | UMMZ 118946 | 000S10731 |
| *Pseudoficimia frontalis* | UMMZ 112512 | 000S10741 |
| *Scolecophis atrocinctus* | UMMZ 131471 | 000S10752 |
| *Scolecophis atrocinctus* | UMMZ 209968 | 000S19961 |
| *Sonora cincta* | UMMZ 137137 | 000S10713 |
| *Sonora occipitalis* | CAS 223594 | 000S18018 |
| *Sonora occipitalis* | UMMZ 116232 | 000S10714 |
| *Sonora semiannulata* | CAS 206503 | 000S18013 |
| *Sonora semiannulata* | UMFS 21015 | 000S15033 |
| *Sonora semiannulata* | UMMZ 21016 | 000S11136 |
| *Sonora straminea* | MVZ 236386 | 000S19241 |
| *Stenorrhina degenhardtii* | UMMZ 124158 | 000S10754 |
| *Stenorrhina freminvillei* | UMMZ 83295 | 000S10755 |
| *Sympholis lippiens* | CAS 132247 | 000S24558 |
| *Tantilla atriceps* | UMMZ 142571 | 0000S9942 |
| *Tantilla bocourti* | UMMZ 143721 | 0000S9941 |
| *Tantilla calamarina* | UMMZ 104499 | 0000S9943 |
| *Tantilla coronata* | UMMZ 152137 | 0000S9940 |
| *Tantilla gracilis* | UMMZ 105282 | 0000S9938 |
| *Tantilla hobartsmithi* | UMMZ 225397 | 0000S9937 |
| *Tantilla jani* | UMMZ 101461 | 000S11127 |
| *Tantilla melanocephala* | UMMZ 60729 | 000S12134 |
| *Tantilla moesta* | UMMZ 79059 | 0000S9936 |
| *Tantilla nigriceps* | UMMZ 69019 | 0000S9944 |
| *Tantilla relicta* | UMMZ 128508 | 0000S9935 |
| *Tantilla rubra* | UMMZ 111046 | 0000S9934 |
| *Tantilla rubra* | UTA R-12455 | **XXXX*** |
| *Tantilla schistosa* | UMMZ 155726 | 0000S9901 |
| *Tantilla shawi* | UTA R-36810 | **XXXX*** |
| *Tantilla tjiasmantoi* | ZFMK 95238 | Koch & Venegas, 2016 |
| *Tantilla vermiformis* | UMMZ 135259 | 0000S9900 |
| *Tantilla wilcoxi* | UMMZ 77242 | 000S11128 |
| *Tantillita lintoni* | UMMZ 117905 | 000S22760 |
| *Yakacoatl tlalli* | MZFC-HE 37100 | ark:/87602/m4/758119 |
| *Yakacoatl tlalli* | UTA R-66192 | ark:/87602/m4/758195 |
