# Supplementary material for: Sticking our nose into the Sonorini tribe: A new genus and species of snake (Squamata: Colubridae: Sonorini) from the Balsas Basin of Mexico
Source: PLoS One. 2025 Dec 10;20(12):e0337187. doi: 10.1371/journal.pone.0337187 (PMC12694871; doi:10.1371/journal.pone.0337187)
Supplement: S2 Table — Taxa in bold represent members of the tribe Sonorini. (DOCX) [file pone.0337187.s004.docx]

**Table S2.**Species, specimen vouchers, and GenBank accession numbers used in our phylogenetic analysis. Taxa in bold represent members of the tribe Sonorini.

| **Family** | **Species** | **Voucher** | **GenBank accession number** | | | |
| --- | --- | --- | --- | --- | --- | --- |
|  |  |  | **12S** | **16S** | **cytb** | **cmos** |
| Acrochordidae | *Acrochordus granulatus* | — | AF544738 | AF544786 | AF217841 | HM234057 |
| Aniliidae | *Anilius scytale* | — | AF544753 | AF544826 | U69738 | AF544722 |
| Atractaspididae | *Atractaspis micropholis* | IPMB J283 | AF544740 | AY611823 | AY612006 | AY611915 |
| Boidae | *Boa constrictor* |  | NC007398 | NC007398 | NC007398 | OK349610 |
| Boidae | *Eryx conicus* | — | GQ225680 | — | GQ225658 | GQ225672 |
| Calamariidae | *Calamaria pavimentata* | ROM 35597 | KX694584 | KX694624 | KX694890 | KX694804 |
| Colubridae | *Bogertophis subocularis* | — | — | — | DQ902103 | DQ902060 |
| Colubridae | *Bogertophis rosaliae* | CAS 173170 | — | — | DQ902102 | DQ902059 |
| Colubridae | *Cemophora coccinea* | CAS 203080 | — | — | AF471091 | AF471132 |
| Colubridae | *Chironius exoletus* | QCAZ 13303 | MK086656 | MK086553 | OK143277 | MK086755 |
| Colubridae | *Coluber constrictor* | ROM 23025 | KX694604 | KX694632 | — | KX694806 |
| Colubridae | ***Conopsis biserialis*** | MZFC 11509 | — | — | GQ895860 | GQ895804 |
| Colubridae | ***Conopsis lineata*** | JAC 22813 | MK209178 | MK209292 | MK209255 | MK209219 |
| Colubridae | ***Conopsis nasus*** | JAC 23430 | MK209179 | — | MK209256 | MK209220 |
| Colubridae | *Dendrophidion dendrophis* | IBUSP 107-18 | MK209180 | MK209293 | MK209257 | — |
| Colubridae | *Drymarchon melanurus* | CTMZ 19586 | — | MK209294 | — | MK209222 |
| Colubridae | *Drymobius chloroticus* | JAC22037 | MK209182 | MK209295 | MK209259 | — |
| Colubridae | *Drymobius rhombifer* | WED59315 | HM565761 | HM582220 | GQ927320 | GQ927313 |
| Colubridae | *Drymoluber brazili* | MZUSP 15507 | MK209184 | MK209296 | MK209260 | MK209223 |
| Colubridae | ***Ficimia publia*** | JAC 22652 | MK209190 | — | MK209265 | MK209224 |
| Colubridae | ***Ficimia streckeri*** | CJF 3846 | — | — | KU859572 | KU859460 |
| Colubridae | ***Gyalopion canum*** | USNM 315524 | MK209191 | MK209300 | MK209266 | MK209225 |
| Colubridae | ***Gyalopion quadrangulare*** | ROM-JRO655 | MK209192 | MK209301 | MK209267 | MK209226 |
| Colubridae | *Lampropeltis getula* | ROM 13727 | KX694603 | KX694649 | KX694866 | KX694811 |
| Colubridae | *Leptodrymus pulcherrimus* | FN 256061 | KR814627 | KR814649 | KR814687 | KR814666 |
| Colubridae | *Leptophis ahaetulla* | MZUSP 18646 | MK209193 | MK209302 | MK209268 | MK209227 |
| Colubridae | *Leptophis diplotropis* | LSUMNS6328 | — | KX660271 | KX660540 | KX660408 |
| Colubridae | *Mastigodryas boddaerti* | MZUSP 19781 | MK209194 | MK209303 | MK209269 | MK209229 |
| Colubridae | *Mastigodryas dorsalis* | KU 289864 | KR814625 | KR814650 | KR814691 | KR814667 |
| Colubridae | *Mastigodryas melanolomus* | JAC 23752 | MK209197 | MK209307 | MK209272 | — |
| Colubridae | *Mastigodryas pulchriceps* | MHNUCS 511 | MK209199 | MK209312 | MK209274 | MK209238 |
| Colubridae | *Masticophis mentovarius* | MVZ 207361 | — | — | MF402826 | MF402240 |
| Colubridae | *Opheodrys aestivus* | CAS 173661 | — | — | MZ080386 | AF471147 |
| Colubridae | *Oxybelis fulgidus* | UTA R-52506 | MT969243 | MT969277 | MK497173 | MK497197 |
| Colubridae | *Oxybelis microphtalmus* | MZFC 19224 | MT969257 | MT969295 | MK497185 | MK497209 |
| Colubridae | *Palusophis bifossatus* | MZUSP 14651 | MK209204 | MK209317 | MK209279 | MK209242 |
| Colubridae | *Phrynonax poecilonotus* | JMR 744 | — | — | KF669672 | KF669706 |
| Colubridae | *Phyllorhynchus decurtatus* | ROM 13682 | — | MK209319 | — | MK209244 |
| Colubridae | *Phyllorhynchus browni* | LSU H-6336 | — | — | KP765666 | KP765644 |
| Colubridae | *Pituophis deppei* | ROM 15332 | KX694582 | KX694657 | KX694867 | KX694814 |
| Colubridae | ***Pseudoficimia frontalis*** | EPR 1453 | MK209206 | MK209320 | MK209280 | MK209245 |
| Colubridae | *Ptyas korros* | ROM 30908 | KX694591 | KX694663 | KX694869 | KX694817 |
| Colubridae | *Rhinobothryum lentiginosum* | CTMZ16062 | MK209207 | MK209322 | MK209282 | MK209247 |
| Colubridae | *Salvadora grahamiae* | LSU H-8715 | — | — | KP765667 | KP765649 |
| Colubridae | *Salvadora hexalepis* | ROM 13548 | MK209208 | MK209323 | MK209283 | MK209248 |
| Colubridae | ***Scolecophis atrocinctus*** | KU 289804 | KR814619 | KR814642 | KR814703 | KR814668 |
| Colubridae | *Simophis rhinostoma* | CTMZ 00076 | MK209210 | — | MK209284 | MK209249 |
| Colubridae | ***Sonora aemula*** | UANL 6976 | — | — | JQ265959 | JQ265952 |
| Colubridae | ***Sonora michoacanensis*** | MZFC 23956 | — | — | JQ265958 | JQ265951 |
| Colubridae | ***Sonora mutabilis*** | UTA-R 53488 | — | — | JQ265953 | JQ265947 |
| Colubridae | ***Sonora occipitalis*** | YPX 513 | MK209211 | MK209325 | — | — |
| Colubridae | ***Sonora semiannulata*** | USNM 315525 | MK209212 | — | MK209285 | MK209250 |
| Colubridae | ***Sonora straminea*** | CTMZ 07550 | MK209213 | MK209326 | MK209286 | — |
| Colubridae | *Spilotes pullatus* | ADM 259 | — | KX660284 | KX660550 | KX660423 |
| Colubridae | *Spilotes sulphureus* | MZUSP 15578 | MK209215 | MK209328 | MK209287 |  |
| Colubridae | ***Stenorrhina degenhardtii*** | CH 5823 | — | MH140988 | — | — |
| Colubridae | ***Stenorrhina freminvillei*** | KU 289794 | — | MK209330 | — | — |
| Colubridae | *Symphimus leucostomus* | MVZ 175960 | KR814618 | KR814651 | KR814690 | KR814670 |
| Colubridae | ***Sympholis lippiens*** | MZFC 10960 | — | — | GQ895890 | GQ895831 |
| Colubridae | ***Tantilla alticola*** | JM590 | — | MH140993 | — | — |
| Colubridae | ***Tantilla armillata*** | FN 256487 | KR814613 | KR814644 | — | KR814681 |
| Colubridae | ***Tantilla berguidoi*** | SMF 97636 | — | Batista et al. 2016 | — | — |
| Colubridae | ***Tantilla boipiranga*** | IBSP 90487 | — | MW627269 | MW627255 | MW627247 |
| Colubridae | ***Tantilla coronata*** | LSUH-18896 | — | — | KP765669 | KP765653 |
| Colubridae | ***Tantilla gracilis*** | OMNH41880 | — | — | KP765670 | KP765654 |
| Colubridae | ***Tantilla hobartsmithi*** | MVZ 233299 | — | — | KP765671 | KP765650 |
| Colubridae | ***Tantilla impensa*** | FN 253542 | KR814614 | KR814645 | KR814688 | KR814677 |
| Colubridae | ***Tantilla melanocephala*** | MZUSP 12976 | MK209216 | MK209331 | MK209288 | — |
| Colubridae | ***Tantilla nigriceps*** | OMNH41890 | — | — | KP765672 | KP765655 |
| Colubridae | ***Tantilla planiceps*** | TAPL 340 | — | — | KP765673 | KP765651 |
| Colubridae | ***Tantilla relicta*** | CAS 200845 | — | — | AF471045 | AF471107 |
| Colubridae | ***Tantilla selmae*** | IBSP 90139 | — | OP422455 | OP432476 | OP432483 |
| Colubridae | ***Tantilla supracincta*** | CH 6094 | — | MH140994 | — | — |
| Colubridae | ***Tantilla tjiasmantoi*** | CORBIDI 7726 | KY006875 | KY006877 | — | — |
| Colubridae | ***Tantilla vermiformis*** | FN 256027 | KR814615 | KR814646 | KR814684 | KR814665 |
| Colubridae | ***Tantilla wilcoxi*** | JAC 29265 | — | — | Cox et al. 2018 | — |
| Colubridae | *Trimorphodon biscutatus* | JAC 24310 | MK209217 | — | MK209289 | MK209254 |
| Colubridae | ***Yakacoatl tlalli*** | MZFC 37100, holotype | PV897249 | PV897250 | PV877222 | PV877223 |
| Colubridae | ***Yakacoatl tlalli*** | UTA-R 66192, paratype | — | PV897251 | PV932163 | PV932164 |
| Dipsadidae | *Atractus atlas* | QCAZ 14946 | — | MH790470 | MN887669 | MN887640 |
| Dipsadidae | *Caaeteboia amarali* | IBSP 72585 | GQ457807 | GQ457747 | JQ598921 | GQ457867 |
| Dipsadidae | *Farancia abacura* | RAP 568 | KR814628 | KR814639 | KR814694 | KR814674 |
| Dipsadidae | *Paikwaophis kruki* | RBINS 2734 | OR075146 | OR075194 | OR069382 | OR069381 |
| Elapidae | *Micrurus diutius* | CAS 231787 | MK534139 | MK534149 | MK534168 | MK534158 |
| Homalopsidae | *Homalopsis buccata* | FMNH 252514 | EF395892 | EF395868 | EF395917 | EF395940 |
| Natricidae | *Rhabdophis subminiatus* | KIZYPX18593 | — | — | MW199836 | MW177864 |
| Pareatidae | *Pareas carinatus* | CAS 247982 | — | — | MZ712233 | MZ712291 |
| Psammophiidae | *Rhamphiophis oxyrhynchus* | ROM 21917 | KX694548 | KX694664 | JQ598953 | KX694825 |
| Pseudoxenodontidae | *Pseudoxenodon karlschmidti* | ROM 41033 | KX694578 | KX694676 | KX694893 | KX694816 |
| Tropidophiidae | *Tropidophis taczanowskyi* | QCAZ 6167 | — | — | KF811126 | KF811112 |
| Xenodermidae | *Xenodermus javanicus* | — | AF544781 | AF544810 | — | AF544711 |
| Xenodermidae | *Stoliczkia vanhnuailianai* | BNHS 3656 | OL352693 | OL352694 | OL422473 | OL422475 |
| Viperidae | *Botriechis rahimi* | MZUTI 3325 | PP213239 | PP214314 | PP249658 | — |
| Viperidae | *Azemiops feae* | ROM 36976 | KX694579 | KX694684 | KX694840 | KX694774 |
