# Supplementary material for: Sticking our nose into the Sonorini tribe: A new genus and species of snake (Squamata: Colubridae: Sonorini) from the Balsas Basin of Mexico
Source: PLoS One. 2025 Dec 10;20(12):e0337187. doi: 10.1371/journal.pone.0337187 (PMC12694871; doi:10.1371/journal.pone.0337187)
Supplement: S3 Table — (DOCX) [file pone.0337187.s005.docx]

**Table S3.**Mexican snake species listed with at least one taxonomic status modification since the year 2000 to date.

| **Species** | **Year of publication** | **New population** | **Resurrection** | **Cryptic lineage** | **Taxonomic revision** | **Habit** | **Reference** |
| --- | --- | --- | --- | --- | --- | --- | --- |
| *Cenaspis aenigma* | 2018 | X |  |  |  | Fossorial | Campbell J. A., Smith E. N., and Hall A. S. 2018. Caudals and calyces: the curious case of a consumed Chiapan Colubroid. Journal of Herpetology 52(4): 459-472. |
| *Chersodromus australis* | 2018 | X |  |  |  | Semifossorial | Canseco-Márquez L., Ramírez-González C. G., and Campbell J. A. 2018. Taxonomic review of the rare Mexican snake genus Chersodromus (Serpentes: Dipsadidae), with the description of two new species. Zootaxa 4399 (2): 151-169. |
| *Chersodromus nigrum* | 2018 | X |  |  |  | Semifossorial | Canseco-Márquez L., Ramírez-González C. G., and Campbell J. A. 2018. Taxonomic review of the rare Mexican snake genus Chersodromus (Serpentes: Dipsadidae), with the description of two new species. Zootaxa 4399 (2): 151-169. |
| *Coniophanes michoacanensis* | 2018 | X |  |  |  | Terrestrial | Flores-Villela O. & Smith E.N. 2009. A new species of Coniophanes (Squamata: Colubridae) from Michoacán, Mexico. Herpetologica 65: 404–412. |
| *Coniophanes taeniatus* | 1870 |  | X |  | X | Terrestrial | Palacios-Aguilar R., & Flores-Villela O. 2020. Taxonomic revision and comments on two groups of the genus Coniophanes (Squamata: Dipsadidae). Vertebrate Zoology 70 (2): 111-124. |
| *Coniophanes taylori* | 1951 |  |  |  | X | Terrestrial | Flores-Villela O. & Smith E.N. 2009. A new species of Coniophanes (Squamata: Colubridae) from Michoacán, Mexico. Herpetologica 65: 404–412. |
| *Conophis morai* | 2002 | X |  |  |  | Terrestrial | Pérez-Higareda, Gonzalo, Marco A. López-Luna and Hobart M. Smith 2002. A new species of Conophis (Reptilia: Serpentes) from Los Tuxtlas, an area of high endemism in southern Veracruz, Mexico. Bulletin of the Maryland Herpological Society 38(1):27-32. |
| *Croatlus armstrongi* | 1979 |  |  | X | X | Terrestrial | Bryson, R. W. Jr., Linkem C. W., Dorcas M. E., Lathrop A., Jones J. M., Alvarado-Díaz J., Grünwald C. I. & Murphy R. W. 2014. Multilocus species delimitation in the Crotalus triseriatus species group (Serpentes: Viperidae: Crotalinae), with the description of two new species. Zootaxa 3826 (3): 475–496. |
| *Crotalus bruneus* | 1978 |  |  | X | X | Terrestrial | Blair, C., R. W. Bryson, C. W. Linkem, D. Lazcano, J. Klicka, J. E. McCormack. 2019. Cryptic diversity in the Mexican highlands: Thousands of UCE loci help illuminate phylogenetic relationships, species limits and divergence times of montane rattlesnakes (Viperidae: Crotalus). Molecular Ecology Resources 19: 349-365. |
| *Crotalus campbelli* | 2014 |  |  | X | X | Terrestrial | Bryson, R. W. Jr., Linkem C. W., Dorcas M. E., Lathrop A., Jones J. M., Alvarado-Díaz J., Grünwald C. I. & Murphy R. W. 2014. Multilocus species delimitation in the Crotalus triseriatus species group (Serpentes: Viperidae: Crotalinae), with the description of two new species. Zootaxa 3826 (3): 475–496. |
| *Crotalus ericsmithi* | 2008 | X |  |  |  | Terrestrial | Campbell, J. A. and Flores-Villela O. 2008. A new long-tailed rattlesnake (Viperidae) from Guerrero, Mexico. Herpetologica 64 (2): 246-257. |
| *Crotalus exiguus* | 1979 |  |  | X | X | Terrestrial | Blair, C., R. W. Bryson, C. W. Linkem, D. Lazcano, J. Klicka, J. E. McCormack. 2019. Cryptic diversity in the Mexican highlands: Thousands of UCE loci help illuminate phylogenetic relationships, species limits and divergence times of montane rattlesnakes (Viperidae: Crotalus). Molecular Ecology Resources 19: 349-365. |
| *Crotalus mictlantecuhtli* | 2020 |  |  | X | X | Terrestrial | Carbajal-Márquez, R. A.; Cedeño-Vázquez J. R., Martínez-Arce A., Neri-Castro E., Machkour- M’rabet S. C. 2020. Accessing cryptic diversity in Neotropical rattlesnakes (Serpentes: Viperidae: Crotalus) with the description of two new species. Zootaxa 4729 (4): 451–481 |
| *Crotalus morulus* | 1952 |  |  |  | X | Terrestrial | Bryson, R. W. Jr., Linkem C. W., Dorcas M. E., Lathrop A., Jones J. M., Alvarado-Díaz J., Grünwald C. I. & Murphy R. W. 2014. Multilocus species delimitation in the Crotalus triseriatus species group (Serpentes: Viperidae: Crotalinae), with the description of two new species. Zootaxa 3826 (3): 475–496. |
| *Crotalus polisi* | 2018 |  |  | X | X | Terrestrial | Meik J. M., Schaack S., Flores-Villela O., and Streicher J. W. 2018. Integrative taxonomy at the nexus of population divergence and speciation in insular speckled rattlesnakes. Journal of Natural History 52: 989-1016. |
| *Crotalus tancitarensis* | 2004 | X |  |  |  | Terrestrial | Alvarado-Diaz, J. & Campbell, J.A. 2004. A new montane rattlesnake (Viperidae) from Michoacán, Mexico. Herpetologica 60 (2): 281-286. |
| *Crotalus thalasophorus* | 2018 |  |  | X | X | Terrestrial | Meik J. M., Schaack S., Flores-Villela O., and Streicher J. W. 2018. Integrative taxonomy at the nexus of population divergence and speciation in insular speckled rattlesnakes. Journal of Natural History 52: 989-1016. |
| *Crotalus tlaloci* | 2014 |  |  | X | X | Terrestrial | Bryson, R. W. Jr., Linkem C. W., Dorcas M. E., Lathrop A., Jones J. M., Alvarado-Díaz J., Grünwald C. I. & Murphy R. W. 2014. Multilocus species delimitation in the Crotalus triseriatus species group (Serpentes: Viperidae: Crotalinae), with the description of two new species. Zootaxa 3826 (3): 475–496. |
| *Epictia resetari* | 2016 |  |  | X | X | Fossorial | Wallach, V. 2016. Morphological review and taxonomic status of the Epictia phenops species group of Mesoamerica, with description of six new species and discussion of South American Epictia albifrons, E. goudotii, and E. tenella (Serpentes: Leptotyphlopidae: Epictinae Mesoamerican Herpetology 3 (2): 216-374. |
| *Epictia schneideri* | 2016 |  |  | X | X | Fossorial | Wallach, V. 2016. Morphological review and taxonomic status of the Epictia phenops species group of Mesoamerica, with description of six new species and discussion of South American Epictia albifrons, E. goudotii, and E. tenella (Serpentes: Leptotyphlopidae: Epictinae Mesoamerican Herpetology 3 (2): 216-374. |
| *Epictia vindumi* | 2016 |  |  | X | X | Fossorial | Wallach, V. 2016. Morphological review and taxonomic status of the Epictia phenops species group of Mesoamerica, with description of six new species and discussion of South American Epictia albifrons, E. goudotii, and E. tenella (Serpentes: Leptotyphlopidae: Epictinae Mesoamerican Herpetology 3 (2): 216-374. |
| *Epictia wynni* | 2016 |  |  | X | X | Fossorial | Wallach, V. 2016. Morphological review and taxonomic status of the Epictia phenops species group of Mesoamerica, with description of six new species and discussion of South American Epictia albifrons, E. goudotii, and E. tenella (Serpentes: Leptotyphlopidae: Epictinae Mesoamerican Herpetology 3 (2): 216-374. |
| *Geophis berillus* | 2022 | X |  |  |  | Fossorial | Barragán-Reséndiz L. M., Pavón-Vázquez C. J., Cervantes-Burgos R. I., Trujano-Ortega M., Canseco-Márquez L. & García-Vázquez U. O. 2022. A New Species of Snake of the Geophis sieboldi Group (Squamata: Dipsadidae) from Estado de México, Mexico. Herpetologica. 78(4); 268-276. |
| *Geophis cansecoi* | 2021 | X |  |  |  | Fossorial | Grünwald CI, Ahumada-Carrillo IT, Grünwald AJ, Montaño-Ruvalcaba CE, García-Vázquez UO. 2021. A new species of Geophis (Dipsadidae) from Veracruz, Mexico, with comments on the validity of related taxa. Amphibian & Reptile Conservation 15(2) [Taxonomy Section]: 289–310 (e294) |
| *Geophis fuscus* | 1886 |  | X |  |  | Fossorial | Grünwald CI, Ahumada-Carrillo IT, Grünwald AJ, Montaño-Ruvalcaba CE, García-Vázquez UO. 2021. A new species of Geophis (Dipsadidae) from Veracruz, Mexico, with comments on the validity of related taxa. Amphibian & Reptile Conservation 15(2) [Taxonomy Section]: 289–310 (e294) |
| *Geophis juarezi* | 2004 | X |  |  |  | Fossorial | Nieto-Montes de Oca, A. 2003. A new species of the Geophis dubius group (Squamata: Colubridae) from the Sierra de Juárez of Oaxaca, Mexico. Herpetologica 59 (4): 572-585. |
| *Geophis juliai* | 2001 | X |  |  |  | Fossorial | Pérez-Higareda, G.; Smith, H.M. & López-Luna, M.A. 2001. A new Geophis (Reptilia: Serpentes) from Southern Veracruz, Mexico. Bulletin of the Maryland Herpological Society 37 (2): 42-48. |
| *Geophis lorancai* | 2016 | X |  |  |  | Fossorial | Canseco-Márquez L., Pavón-Vázquez C. J., López-Luna M. A., and Nieto-Montes de Oca A. 2016. A new species of earth snake (Dipsadidae, Geophis) from Mexico. ZooKeys 610: 131-145. |
| *Geophis occabus* | 2011 | X |  |  |  | Fossorial | Pavón-Vázquez C. J., García-Vázquez U. O., Blancas-Hernández J. C., and Nieto-Montes de Oca A. 2011. A new species of the Geophis sieboldi group (Squamata: Colubridae) exhibiting color pattern polymorphism from Guerrero, Mexico. Herpetologica 67(3): 332-343. |
| *Geophis turbidus* | 2013 | X |  |  |  | Fossorial | Pavón-Vázquez C. J., Canseco-Márquez L., and Nieto-Montes de Oca A. 2013. A new species in the Geophis dubius group (Squamata: Colubridae) from northern Puebla. Mexico. Herpetologica 69(3): 358-370. |
| *Lampropeltis webbi* | 2005 | X |  |  |  | Terrestrial | Bryson, R.W.; Dixon, J.R. & Lazcano, D. 2005. New species of Lampropeltis (Serpentes: Colubridae) from the Sierra Madre Occidental, México. Journal of Herpetology 39 (2): 207-214. |
| *Metlalpicoatlus borealis* | 2021 | X |  |  |  | Terrestrial | Tepos-Ramírez M., Flores-Villela O., Velasco J. A., Pedraza Lara C., García Rubio O. R. & Jadin R. C. 2021. Molecular Phylogenetics and Morphometrics Reveal a New Endemic Jumping Pitviper (Serpentes: Viperidae: *Metlapilcoatlus*) from the Sierra Madre Oriental of Mexico. Journal of Herpetology 55 (2): 181-191. |
| *Mixcoatlus browni* | 1938 |  | X |  | X | Terrestrial | Jadin, R. C.; E. N. Smith and J. A. Campbell. 2011. Unravelling a tangle of Mexican serpents: a systematic revision of highland pitvipers. Zoological Journal of the Linnean Society 163: 943–958 |
| *Ophryacus smaragdinus* | 2015 |  |  | X | X | Arbustive | Grünwald C. I., Jones J. M., Franz-Chávez H., and Ahumada-Carrillo I. T. 2015. A new species of Ophryacus (Serpentes: Viperidae: Crotalinae) from eastern Mexico, with comments on the taxonomy of related pitvipers. Mesoamerican Herpetology 2: 388-416. |
| *Ophryacus sphenophrys* | 1960 |  | X |  | X | Arbustive | Grünwald C. I., Jones J. M., Franz-Chávez H., and Ahumada-Carrillo I. T. 2015. A new species of Ophryacus (Serpentes: Viperidae: Crotalinae) from eastern Mexico, with comments on the taxonomy of related pitvipers. Mesoamerican Herpetology 2: 388-416. |
| *Oxybelis microphtalmus* | 1926 |  | X |  | X | Arboreal | Jadin R. C., Blair C., Orlofske S. A., Jowers M. J., Rivas G. A., Vitt L. J., Ray J. M., Smith E. N. & Murphy J. C. 2020. Not withering on the evolutionary vine: systematic revision of the Brown Vine Snake (Reptilia: Squamata: Oxybelis) from its northern distribution. Organisms Diversity and Evolution 20: 723-746. |
| *Oxybelis potosiensis* | 1941 |  | X |  | X | Arboreal | Jadin R. C., Blair C., Orlofske S. A., Jowers M. J., Rivas G. A., Vitt L. J., Ray J. M., Smith E. N. & Murphy J. C. 2020. Not withering on the evolutionary vine: systematic revision of the Brown Vine Snake (Reptilia: Squamata: Oxybelis) from its northern distribution. Organisms Diversity and Evolution 20: 723-746. |
| *Rena klauberi* | 2022 | X |  |  |  | Fossorial | Flores-Villela, O. A., Smith, E. N., Canseco-Márquez, L., & Campbell, J. A. 2022. A new species of blindsnake from Jalisco, Mexico (Squamata: Leptotyphlopidae). Revista Mexicana de Biodiversidad, 93: 933933 |
| *Rhadinaea nuchalis* | 2018 | X |  |  |  | Semifossorial | García-Vázquez U. O., Pavón-Vázquez C. J., Blancas-Hernández J. C., Blancas-Calva E. & Centenero-Alcalá E. 2018. A new rare species of the Rhadinaea decorata group from the Sierra Madre del Sur of Guerrero, Mexico (Squamata, Colubridae). ZooKeys 780: 137–154. |
| *Rhadinella donaji* | 2015 | X |  |  |  | Semifossorial | Campbell, J. A. 2015. A new species of Rhadinella (Serpentes: Colubridae) from the Pacific versant of Oaxaca, Mexico. Zootaxa 3918 (3): 397–405 |
| *Rhadinella dysmica* | 2016 | X |  |  |  | Semifossorial | Campillo G., Dávila-Galavíz L. F., Flores-Villela O., and Campbell J. A. 2016. A new species of Rhadinella (Serpentes: Colubridae) from the Sierra Madre del Sur of Guerrero, Mexico. Zootaxa 4103 (2): 165-173. |
| *Salvadora gymnorachis* | 2019 | X |  |  |  | Terrestrial | Hernández-Jiménez C. A., Flores-Villela O. A., and Campbell J. A. 2019. A new species of patch-nosed snake (Colubridae: Salvadora Baird and Girard, 1853) from Oaxaca, Mexico. Zootaxa 4564(2): 588-600. |
| *Sibon linearis* | 2002 | X |  |  |  | Terrestrial | Pérez-Higareda, G., López-Luna M. A., & Smith H. M. 2002. A new snake related to Sibon sanniola (Serpentes: Dipsadidae) from Los Tuxtlas, Veracruz, Mexico. Bulletin of the Maryland Herpological Society 38(2):62-65. |
| *Tantilla carolina* | 2022 | X |  |  |  | Semifossorial | Palacios-Aguilar, R., Fucsko, L. A., Jiménez-Arcos, V. H., Wilson, L. D., & Mata-Silva, V. 2022. Out of the Past: A new species of Tantilla of the calamarina group (Squamata: Colubridae) from southeastern coastal Guerrero, Mexico, with comments on relationships within members of the group. Amphibian & Reptile Conservation, 16(2): 120-132 |
| *Tantilla ceboruca* | 2007 | X |  |  |  | Semifossorial | Canseco-Márquez, L.; E. N. Smith, P. Ponce-Campos, O. Flores-Villela, J. A. Campbell 2007. A new species of Tantilla (Squamata: Colubridae) of the calamarina group from Volcán Ceboruco, Nayarit, Mexico. Journal of Herpetology 41 (2): 220-224 |
| *Tantilla robusta* | 2002 | X |  |  |  | Semifossorial | Canseco-Márquez, L.; Mendelson III, J.R. & Gutiérrez-Mayén, G. 2002. A new species of large Tantilla (Squamata: Colubridae) from the Sierra Madre Oriental of Puebla, Mexico. Herpetologica 58 (4): 492-497. |
| *Tantilla sertula* | 2000 | X |  |  |  | Semifossorial | Wilson L. D. & Campbell J. A. 2000. A new species of the calamarina group of the colubrid snake genus Tantilla (Reptilia: Squamata) from Guerrero, Mexico, with a review of and key to members of the group. Proceedings of the Biological Society of Washington 113 (3): 820-827 |
| *Thamnophis ahumadai* | 2024 |  |  |  | X | Terrestrial | Grünwald C. I., Mendoza-Portilla M. C. G., Grünwald A. J., Montaño-Ruvalcaba C., Franz-Chávez H., García-Vázquez U. O. & Reyes-Velasco J. 2024. A new species of *Thamnophis* (Serpentes, Colubridae) from Jalisco, Mexico, and distribution of snakes related to *Thamnophis scalaris*. Herpetozoa 37: 157-179. |
| *Thamnophis bogerti* | 2005 |  |  | X | X | Terrestrial | Rossman, D.A. & Burbrink, F.T. 2005. Species limits within the Mexican garter snakes of the Thamnophis godmani complex. Occasional Papers of the Museum of Natural Science (79): 1-43 |
| *Thamnophis conanti* | 2005 |  |  | X | X | Terrestrial | Rossman, D.A. & Burbrink, F.T. 2005. Species limits within the Mexican garter snakes of the Thamnophis godmani complex. Occasional Papers of the Museum of Natural Science (79): 1-44 |
| *Thamnophis lineri* | 2005 |  |  | X | X | Terrestrial | Rossman, D.A. & Burbrink, F.T. 2005. Species limits within the Mexican garter snakes of the Thamnophis godmani complex. Occasional Papers of the Museum of Natural Science (79): 1-45 |
| *Thamnophis rossmani* | 2000 | X |  |  |  | Terrestrial | Conant R. 2000. A new species of garter snake from Western Mexico. Occasional Papers of the Museum of Natural Science, Louisiana State University, Baton Rouge, No. 76: 1-7 |
| *Tropidodipsas guerreroensis* | 1939 |  |  |  | X | Arboreal | Grünwald CI, Toribio-Jiménez S, Montaño-Ruvalcaba C, Franz-Chávez H, Peñaloza-Montaño MA, Barrera-Nava EY, Jones JM, Rodriguez CM, Hughes IM, Strickland JL, Reyes-Velasco J 2021. Two new species of snail-eating snakes of the genus Tropidodipsas (Serpentes, Dipsadidae) from southern Mexico, with notes on related species. Herpetozoa 34: 233-257 |
| *Tropidodipsas papavericola* | 2021 | X |  |  |  | Arboreal | Grünwald CI, Toribio-Jiménez S, Montaño-Ruvalcaba C, Franz-Chávez H, Peñaloza-Montaño MA, Barrera-Nava EY, Jones JM, Rodriguez CM, Hughes IM, Strickland JL, Reyes-Velasco J 2021. Two new species of snail-eating snakes of the genus Tropidodipsas (Serpentes, Dipsadidae) from southern Mexico, with notes on related species. Herpetozoa 34: 233-258 |
| *Tropidodipsas repleta* | 2005 | X |  |  |  | Terrestrial | Smith H. M.; Lemos-Espinal J. A.; Hartman D. & Chiszar D. 2005. A new species of Tropidodipsas (Serpentes: Colubridae) from Sonora, Mexico. Bulletin of the Maryland Herpological Society 41: 39-41 |
| *Tropidodipsas tricolor* | 2021 | X |  |  |  | Arboreal | Grünwald CI, Toribio-Jiménez S, Montaño-Ruvalcaba C, Franz-Chávez H, Peñaloza-Montaño MA, Barrera-Nava EY, Jones JM, Rodriguez CM, Hughes IM, Strickland JL, Reyes-Velasco J 2021. Two new species of snail-eating snakes of the genus Tropidodipsas (Serpentes, Dipsadidae) from southern Mexico, with notes on related species. Herpetozoa 34: 233-258 |
| *Yakacoatl tlalli* | 2024 | X |  |  |  | Semifossorial | This paper |
