# Supplementary material for: Sticking our nose into the Sonorini tribe: A new genus and species of snake (Squamata: Colubridae: Sonorini) from the Balsas Basin of Mexico
Source: PLoS One. 2025 Dec 10;20(12):e0337187. doi: 10.1371/journal.pone.0337187 (PMC12694871; doi:10.1371/journal.pone.0337187)
Supplement: S4 Table — (DOCX) [file pone.0337187.s006.docx]

**Table S4.** Genetic uncorrected pairwise distances calculated from the 12S gene using MEGA X software.

|  | ***Pseudoficimia***  ***frontalis*** | ***Yakacoatl tlalli* holotype** | ***Conopsis***  ***lineata*** | ***Conopsis***  ***nasus*** | ***Sonora***  ***semiannulata*** | ***Sonora***  ***straminea*** | ***Sonora***  ***occipitalis*** | ***Ficimia***  ***publia*** | ***Gyalopion***  ***canum*** | ***Gyalopion***  ***quadrangulare*** | ***Tantilla***  ***armillata*** | ***Tantilla***  ***impensa*** | ***Tantilla***  ***melanocephala*** | ***Tantilla***  ***tjiasmantoi*** | ***Tantilla***  ***vermiformis*** | ***Scolecophis***  ***atrocinctus*** |
| --- | --- | --- | --- | --- | --- | --- | --- | --- | --- | --- | --- | --- | --- | --- | --- | --- |
| ***Pseudoficimia***  ***frontalis*** |  |  |  |  |  |  |  |  |  |  |  |  |  |  |  |  |
| ***Yakacoatl***  ***tlalli* holotype** | 0.1061 |  |  |  |  |  |  |  |  |  |  |  |  |  |  |  |
| ***Conopsis***  ***lineata*** | 0.1098 | 0.1326 |  |  |  |  |  |  |  |  |  |  |  |  |  |  |
| ***Conopsis***  ***nasus*** | 0.1212 | 0.1288 | 0.0606 |  |  |  |  |  |  |  |  |  |  |  |  |  |
| ***Sonora***  ***semiannulata*** | 0.1212 | 0.1402 | 0.1250 | 0.1212 |  |  |  |  |  |  |  |  |  |  |  |  |
| ***Sonora***  ***straminea*** | 0.1212 | 0.1364 | 0.1174 | 0.1212 | 0.0682 |  |  |  |  |  |  |  |  |  |  |  |
| ***Sonora***  ***occipitalis*** | 0.1288 | 0.1402 | 0.1364 | 0.1477 | 0.0871 | 0.0871 |  |  |  |  |  |  |  |  |  |  |
| ***Ficimia***  ***publia*** | 0.1136 | 0.1326 | 0.0947 | 0.1174 | 0.0985 | 0.1023 | 0.1136 |  |  |  |  |  |  |  |  |  |
| ***Gyalopion***  ***canum*** | 0.1288 | 0.1288 | 0.1174 | 0.1250 | 0.1326 | 0.1212 | 0.1326 | 0.1212 |  |  |  |  |  |  |  |  |
| ***Gyalopion***  ***quadrangulare*** | 0.1061 | 0.1250 | 0.1023 | 0.1023 | 0.1061 | 0.0947 | 0.1212 | 0.0985 | 0.0795 |  |  |  |  |  |  |  |
| ***Tantilla***  ***armillata*** | 0.1477 | 0.1515 | 0.1364 | 0.1326 | 0.1250 | 0.1023 | 0.1515 | 0.1250 | 0.1553 | 0.1212 |  |  |  |  |  |  |
| ***Tantilla***  ***impensa*** | 0.1212 | 0.1553 | 0.1250 | 0.1364 | 0.1174 | 0.1098 | 0.1439 | 0.1174 | 0.1288 | 0.1098 | 0.0985 |  |  |  |  |  |
| ***Tantilla***  ***melanocephala*** | 0.1402 | 0.1515 | 0.1364 | 0.1326 | 0.1023 | 0.1212 | 0.1439 | 0.1098 | 0.1591 | 0.1326 | 0.0871 | 0.0985 |  |  |  |  |
| ***Tantilla***  ***tjiasmantoi*** | 0.1553 | 0.1364 | 0.1477 | 0.1439 | 0.1136 | 0.1023 | 0.1439 | 0.1250 | 0.1591 | 0.1212 | 0.0833 | 0.1174 | 0.0682 |  |  |  |
| ***Tantilla***  ***vermiformis*** | 0.1288 | 0.1742 | 0.1439 | 0.1477 | 0.1250 | 0.1212 | 0.1515 | 0.1212 | 0.1288 | 0.1212 | 0.0871 | 0.1061 | 0.1136 | 0.1023 |  |  |
| ***Scolecophis***  ***atrocinctus*** | 0.1212 | 0.1136 | 0.1061 | 0.1136 | 0.0947 | 0.0985 | 0.1288 | 0.0947 | 0.1023 | 0.0758 | 0.0947 | 0.0758 | 0.0833 | 0.1023 | 0.1061 |  |
