# Supplementary material for: Sticking our nose into the Sonorini tribe: A new genus and species of snake (Squamata: Colubridae: Sonorini) from the Balsas Basin of Mexico
Source: PLoS One. 2025 Dec 10;20(12):e0337187. doi: 10.1371/journal.pone.0337187 (PMC12694871; doi:10.1371/journal.pone.0337187)
Supplement: S5 Table — (DOCX) [file pone.0337187.s007.docx]

**Table S5.** Genetic uncorrected pairwise distances calculated from the 16S gene using MEGA X software.

|  | ***Tantilla***  ***berguidoi*** | ***Pseudoficimia***  ***frontalis*** | ***Yakacoatl***  ***tlalli holotype*** | ***Yakacoatl***  ***tlalli paratype*** | ***Stenorrhina***  ***degenhardtii*** | ***Stenorrhina***  ***freminvillei*** | ***Tantilla***  ***boipiranga*** | ***Tantilla***  ***selmae*** | ***Tantilla***  ***melanocephala*** | ***Tantilla***  ***tjiasmantoi*** | ***Tantilla***  ***vermiformis*** | ***Tantilla***  ***impensa*** | ***Tantilla***  ***supracincta*** | ***Tantilla***  ***alticola*** | ***Tantilla***  ***armillata*** | ***Conopsis***  ***lineata*** | ***Sonora***  ***straminea*** | ***Sonora***  ***occipitalis*** | ***Scolecophis***  ***atrocinctus*** | ***Gyalopion***  ***canum*** | ***Gyalopion***  ***quadrangulare*** |
| --- | --- | --- | --- | --- | --- | --- | --- | --- | --- | --- | --- | --- | --- | --- | --- | --- | --- | --- | --- | --- | --- |
| ***Tantilla***  ***berguidoi*** |  |  |  |  |  |  |  |  |  |  |  |  |  |  |  |  |  |  |  |  |  |
| ***Pseudoficimia***  ***frontalis*** | 0.1194 |  |  |  |  |  |  |  |  |  |  |  |  |  |  |  |  |  |  |  |  |
| ***Yakacoatl***  ***tlalli holotype*** | 0.1083 | 0.0628 |  |  |  |  |  |  |  |  |  |  |  |  |  |  |  |  |  |  |  |
| ***Yakacoatl***  ***tlalli paratype*** | 0.1083 | 0.0628 | 0.0000 |  |  |  |  |  |  |  |  |  |  |  |  |  |  |  |  |  |  |
| ***Stenorrhina***  ***degenhardtii*** | 0.1064 | 0.0785 | 0.0709 | 0.0709 |  |  |  |  |  |  |  |  |  |  |  |  |  |  |  |  |  |
| ***Stenorrhina***  ***freminvillei*** | 0.1083 | 0.0754 | 0.0704 | 0.0704 | 0.0152 |  |  |  |  |  |  |  |  |  |  |  |  |  |  |  |  |
| ***Tantilla***  ***boipiranga*** | 0.0917 | 0.0930 | 0.0779 | 0.0779 | 0.0861 | 0.0779 |  |  |  |  |  |  |  |  |  |  |  |  |  |  |  |
| ***Tantilla***  ***selmae*** | 0.0972 | 0.1005 | 0.0879 | 0.0879 | 0.0835 | 0.0804 | 0.0226 |  |  |  |  |  |  |  |  |  |  |  |  |  |  |
| ***Tantilla***  ***melanocephala*** | 0.0861 | 0.0955 | 0.0854 | 0.0854 | 0.0835 | 0.0804 | 0.0176 | 0.0201 |  |  |  |  |  |  |  |  |  |  |  |  |  |
| ***Tantilla***  ***tjiasmantoi*** | 0.0778 | 0.0930 | 0.0804 | 0.0804 | 0.0785 | 0.0804 | 0.0578 | 0.0578 | 0.0528 |  |  |  |  |  |  |  |  |  |  |  |  |
| ***Tantilla***  ***vermiformis*** | 0.0861 | 0.0854 | 0.0729 | 0.0729 | 0.0532 | 0.0553 | 0.0653 | 0.0653 | 0.0603 | 0.0503 |  |  |  |  |  |  |  |  |  |  |  |
| ***Tantilla***  ***impensa*** | 0.0944 | 0.1055 | 0.0879 | 0.0879 | 0.0810 | 0.0905 | 0.0754 | 0.0729 | 0.0729 | 0.0704 | 0.0578 |  |  |  |  |  |  |  |  |  |  |
| ***Tantilla***  ***supracincta*** | 0.0944 | 0.0930 | 0.0829 | 0.0829 | 0.0785 | 0.0829 | 0.0729 | 0.0754 | 0.0704 | 0.0653 | 0.0477 | 0.0251 |  |  |  |  |  |  |  |  |  |
| ***Tantilla***  ***alticola*** | 0.0500 | 0.1005 | 0.0930 | 0.0930 | 0.0937 | 0.0930 | 0.0678 | 0.0729 | 0.0628 | 0.0603 | 0.0553 | 0.0553 | 0.0528 |  |  |  |  |  |  |  |  |
| ***Tantilla***  ***armillata*** | 0.0861 | 0.0905 | 0.0854 | 0.0854 | 0.0810 | 0.0779 | 0.0754 | 0.0754 | 0.0704 | 0.0628 | 0.0729 | 0.0829 | 0.0704 | 0.0779 |  |  |  |  |  |  |  |
| ***Conopsis***  ***lineata*** | 0.1167 | 0.0854 | 0.0779 | 0.0779 | 0.0937 | 0.0930 | 0.0980 | 0.1080 | 0.1055 | 0.1055 | 0.1080 | 0.1106 | 0.1131 | 0.1055 | 0.0955 |  |  |  |  |  |  |
| ***Sonora***  ***straminea*** | 0.1222 | 0.0955 | 0.0854 | 0.0854 | 0.0861 | 0.0905 | 0.0930 | 0.0879 | 0.0930 | 0.0854 | 0.0879 | 0.0804 | 0.0879 | 0.0930 | 0.0980 | 0.0980 |  |  |  |  |  |
| ***Sonora***  ***occipitalis*** | 0.1111 | 0.1080 | 0.1106 | 0.1106 | 0.0911 | 0.0980 | 0.1005 | 0.1005 | 0.1030 | 0.0829 | 0.1005 | 0.0779 | 0.0930 | 0.0905 | 0.0930 | 0.0955 | 0.0804 |  |  |  |  |
| ***Scolecophis***  ***atrocinctus*** | 0.1105 | 0.0997 | 0.0895 | 0.0895 | 0.0851 | 0.0844 | 0.0895 | 0.0895 | 0.0946 | 0.0767 | 0.0895 | 0.0818 | 0.0870 | 0.0921 | 0.0844 | 0.0946 | 0.0870 | 0.0818 |  |  |  |
| ***Gyalopion***  ***canum*** | 0.1111 | 0.0829 | 0.0628 | 0.0628 | 0.0810 | 0.0829 | 0.0905 | 0.0930 | 0.0879 | 0.0854 | 0.0854 | 0.0930 | 0.0804 | 0.0879 | 0.0905 | 0.0930 | 0.0854 | 0.0980 | 0.0767 |  |  |
| ***Gyalopion***  ***quadrangulare*** | 0.1000 | 0.0879 | 0.0553 | 0.0553 | 0.0633 | 0.0578 | 0.0879 | 0.0854 | 0.0905 | 0.0779 | 0.0704 | 0.0804 | 0.0729 | 0.0804 | 0.0879 | 0.0930 | 0.0854 | 0.0854 | 0.0614 | 0.0377 |  |
