# Supplementary material for: Sticking our nose into the Sonorini tribe: A new genus and species of snake (Squamata: Colubridae: Sonorini) from the Balsas Basin of Mexico
Source: PLoS One. 2025 Dec 10;20(12):e0337187. doi: 10.1371/journal.pone.0337187 (PMC12694871; doi:10.1371/journal.pone.0337187)
Supplement: S6 Table — (DOCX) [file pone.0337187.s008.docx]

**Table S6.**Genetic uncorrected pairwise distances calculated from the cytb gene using MEGA X software.

|  | ***Tantilla***  ***boipiranga*** | ***Tantilla***  ***impensa*** | ***Tantilla***  ***melanocephala*** | ***Tantilla***  ***planiceps*** | ***Tantilla***  ***vermiformis*** | ***Tantilla***  ***selmae*** | ***Tantilla***  ***coronata*** | ***Tantilla***  ***gracilis*** | ***Tantilla***  ***hobartsmithi*** | ***Tantilla***  ***nigriceps*** | ***Tantilla***  ***relicta*** | ***Tantilla***  ***wilcoxi*** | ***Sympholis***  ***lippiens*** | ***Ficimia***  ***publia*** | ***Ficimia***  ***streckeri*** | ***Conopsis biserialis*** | ***Conopsis lineata*** | ***Conopsis nasus*** |
| --- | --- | --- | --- | --- | --- | --- | --- | --- | --- | --- | --- | --- | --- | --- | --- | --- | --- | --- |
| ***Tantilla boipiranga*** |  |  |  |  |  |  |  |  |  |  |  |  |  |  |  |  |  |  |
| ***Tantilla impensa*** | 0.15876 |  |  |  |  |  |  |  |  |  |  |  |  |  |  |  |  |  |
| ***Tantilla melanocephala*** | 0.04197 | 0.15511 |  |  |  |  |  |  |  |  |  |  |  |  |  |  |  |  |
| ***Tantilla planiceps*** | 0.16788 | 0.15328 | 0.16058 |  |  |  |  |  |  |  |  |  |  |  |  |  |  |  |
| ***Tantilla vermiformis*** | 0.15693 | 0.16241 | 0.16058 | 0.16423 |  |  |  |  |  |  |  |  |  |  |  |  |  |  |
| ***Tantilla selmae*** | 0.05657 | 0.14234 | 0.04380 | 0.16971 | 0.14964 |  |  |  |  |  |  |  |  |  |  |  |  |  |
| ***Tantilla coronata*** | 0.15511 | 0.12409 | 0.14781 | 0.14416 | 0.15876 | 0.14599 |  |  |  |  |  |  |  |  |  |  |  |  |
| ***Tantilla gracilis*** | 0.14599 | 0.14599 | 0.14781 | 0.10584 | 0.14599 | 0.15146 | 0.13139 |  |  |  |  |  |  |  |  |  |  |  |
| ***Tantilla hobartsmithi*** | 0.15328 | 0.14234 | 0.14781 | 0.12409 | 0.15328 | 0.15328 | 0.12774 | 0.06022 |  |  |  |  |  |  |  |  |  |  |
| ***Tantilla nigriceps*** | 0.16058 | 0.13139 | 0.15876 | 0.11131 | 0.16606 | 0.16241 | 0.11679 | 0.10036 | 0.09672 |  |  |  |  |  |  |  |  |  |
| ***Tantilla relicta*** | 0.14964 | 0.14051 | 0.14416 | 0.13321 | 0.16058 | 0.14416 | 0.04927 | 0.12409 | 0.12956 | 0.11861 |  |  |  |  |  |  |  |  |
| ***Tantilla wilcoxi*** | 0.13504 | 0.14234 | 0.14416 | 0.15146 | 0.14416 | 0.14234 | 0.13869 | 0.12956 | 0.13686 | 0.12956 | 0.12774 |  |  |  |  |  |  |  |
| ***Sympholis lippiens*** | 0.17701 | 0.18978 | 0.17883 | 0.18978 | 0.16606 | 0.17518 | 0.16788 | 0.16788 | 0.15693 | 0.16241 | 0.17153 | 0.17153 |  |  |  |  |  |  |
| ***Ficimia publia*** | 0.21533 | 0.19708 | 0.21350 | 0.17518 | 0.17883 | 0.20803 | 0.20255 | 0.18066 | 0.17336 | 0.16606 | 0.19708 | 0.18796 | 0.15511 |  |  |  |  |  |
| ***Ficimia streckeri*** | 0.20073 | 0.19161 | 0.19708 | 0.17518 | 0.16423 | 0.19891 | 0.19343 | 0.16423 | 0.15693 | 0.16606 | 0.18978 | 0.18248 | 0.15328 | 0.08394 |  |  |  |  |
| ***Conopsis biserialis*** | 0.18248 | 0.18796 | 0.17883 | 0.19161 | 0.16788 | 0.17336 | 0.17336 | 0.16241 | 0.13869 | 0.16241 | 0.17153 | 0.18431 | 0.13504 | 0.15876 | 0.14964 |  |  |  |
| ***Conopsis lineata*** | 0.18248 | 0.20073 | 0.18978 | 0.19343 | 0.17336 | 0.18248 | 0.16788 | 0.16788 | 0.15328 | 0.17153 | 0.16423 | 0.17701 | 0.13504 | 0.16058 | 0.16058 | 0.10219 |  |  |
| ***Conopsis nasus*** | 0.16423 | 0.16058 | 0.16606 | 0.16423 | 0.17153 | 0.16788 | 0.15511 | 0.13869 | 0.12774 | 0.13869 | 0.15511 | 0.17153 | 0.14051 | 0.14964 | 0.14051 | 0.09124 | 0.10949 |  |
| ***Sonora aemula*** | 0.17336 | 0.17701 | 0.16606 | 0.16606 | 0.17518 | 0.16788 | 0.17336 | 0.14964 | 0.14781 | 0.14781 | 0.16241 | 0.15693 | 0.17336 | 0.18066 | 0.17518 | 0.14416 | 0.18431 | 0.14599 |
| ***Sonora mutabilis*** | 0.18613 | 0.18248 | 0.19343 | 0.19161 | 0.18613 | 0.19161 | 0.19343 | 0.16971 | 0.17701 | 0.17518 | 0.18796 | 0.18066 | 0.18978 | 0.16971 | 0.15511 | 0.18431 | 0.19526 | 0.16241 |
| ***Sonora michoacanensis*** | 0.18613 | 0.17518 | 0.18248 | 0.18431 | 0.19161 | 0.18431 | 0.16058 | 0.16423 | 0.14964 | 0.16788 | 0.16788 | 0.18248 | 0.17518 | 0.19161 | 0.16241 | 0.18066 | 0.18613 | 0.15146 |
| ***Sonora straminea*** | 0.16241 | 0.18248 | 0.16788 | 0.17518 | 0.17883 | 0.16241 | 0.18613 | 0.14234 | 0.16606 | 0.15876 | 0.17701 | 0.18248 | 0.15693 | 0.17701 | 0.17518 | 0.17883 | 0.18248 | 0.15693 |
| ***Sonora semiannulata*** | 0.16971 | 0.17701 | 0.17153 | 0.17701 | 0.15876 | 0.15876 | 0.17518 | 0.15511 | 0.15511 | 0.17883 | 0.16606 | 0.16971 | 0.18066 | 0.17701 | 0.16606 | 0.16606 | 0.18431 | 0.15511 |
| ***Gyalopion canum*** | 0.17701 | 0.16606 | 0.18066 | 0.17883 | 0.17701 | 0.17518 | 0.16423 | 0.16241 | 0.15693 | 0.16241 | 0.16058 | 0.18978 | 0.14599 | 0.15146 | 0.14234 | 0.14599 | 0.14234 | 0.14051 |
| ***Pseudoficimia frontalis*** | 0.18248 | 0.18431 | 0.19161 | 0.18796 | 0.17336 | 0.17883 | 0.18248 | 0.18431 | 0.16971 | 0.17336 | 0.17153 | 0.17701 | 0.15511 | 0.17883 | 0.18066 | 0.16241 | 0.17336 | 0.15693 |
| ***Yakacoatl tlalli paratype*** | 0.17269 | 0.16667 | 0.17068 | 0.17871 | 0.18072 | 0.16667 | 0.17671 | 0.17470 | 0.17470 | 0.16667 | 0.17470 | 0.18273 | 0.13855 | 0.14659 | 0.16466 | 0.16466 | 0.16466 | 0.14659 |
| ***Yakacoatl tlalli holotype*** | 0.17701 | 0.17701 | 0.18066 | 0.18066 | 0.17883 | 0.17518 | 0.17336 | 0.17518 | 0.17518 | 0.16606 | 0.17153 | 0.18431 | 0.13504 | 0.14234 | 0.15876 | 0.15693 | 0.16058 | 0.14416 |
| ***Gyalopion quadrangulare*** | 0.18431 | 0.16971 | 0.18066 | 0.16606 | 0.16971 | 0.17518 | 0.15511 | 0.15876 | 0.15511 | 0.15511 | 0.14964 | 0.16606 | 0.12591 | 0.12591 | 0.12956 | 0.14781 | 0.15693 | 0.14781 |
| ***Scolecophis atrocinctus*** | 0.18415 | 0.18844 | 0.18201 | 0.18844 | 0.20128 | 0.19058 | 0.20557 | 0.19272 | 0.19272 | 0.17131 | 0.19486 | 0.20557 | 0.18415 | 0.18415 | 0.16916 | 0.18415 | 0.20343 | 0.17987 |

**S3 Supplementary tables. Table S6** continue.

|  | ***Sonora aemula*** | ***Sonora mutabilis*** | ***Sonora michoacanensis*** | ***Sonora straminea*** | ***Sonora semiannulata*** | ***Gyalopion canum*** | ***Pseudoficimia frontalis*** | ***Yakacoatl tlalli paratype*** | ***Yakacoatl tlalli holotype*** | ***Gyalopion quadrangulare*** | ***Scolecophis atrocinctus*** |
| --- | --- | --- | --- | --- | --- | --- | --- | --- | --- | --- | --- |
| ***Sonora mutabilis*** | 0.15511 |  |  |  |  |  |  |  |  |  |  |
| ***Sonora michoacanensis*** | 0.14781 | 0.13504 |  |  |  |  |  |  |  |  |  |
| ***Sonora straminea*** | 0.14964 | 0.14781 | 0.15511 |  |  |  |  |  |  |  |  |
| ***Sonora semiannulata*** | 0.13321 | 0.16241 | 0.15876 | 0.14964 |  |  |  |  |  |  |  |
| ***Gyalopion canum*** | 0.16788 | 0.15693 | 0.15328 | 0.14599 | 0.14781 |  |  |  |  |  |  |
| ***Pseudoficimia frontalis*** | 0.17153 | 0.17153 | 0.18613 | 0.17883 | 0.15328 | 0.14416 |  |  |  |  |  |
| ***Yakacoatl tlalli paratype*** | 0.16667 | 0.17269 | 0.15462 | 0.16064 | 0.16265 | 0.12249 | 0.15462 |  |  |  |  |
| ***Yakacoatl tlalli holotype*** | 0.17153 | 0.17153 | 0.16241 | 0.16241 | 0.16241 | 0.12044 | 0.16058 | 0.00000 |  |  |  |
| ***Gyalopion quadrangulare*** | 0.16058 | 0.15876 | 0.15693 | 0.15511 | 0.15511 | 0.11314 | 0.13321 | 0.13253 | 0.13504 |  |  |
| ***Scolecophis atrocinctus*** | 0.17773 | 0.18630 | 0.17559 | 0.16916 | 0.16702 | 0.17559 | 0.19058 | 0.18225 | 0.17987 | 0.18201 |  |
